# Gram‐Scale Preparation of Tri‐Coordinated Single‐Atom Catalysts for CO_2_ Electrolysis in Large‐Scale Membrane Electrode Assembly

**DOI:** 10.1002/advs.202500368

**Published:** 2025-03-16

**Authors:** Lei Yuan, Xin Li, Guilin Li, Kuilin Peng, Hongyu Zhang, Shaojuan Zeng, Xiaofu Sun, Xiangping Zhang

**Affiliations:** ^1^ Beijing Key Laboratory of Ionic Liquids Clean Process State Key Laboratory of Multiphase Complex Systems Key Laboratory of Green Process and Engineering Institute of Process Engineering Chinese Academy of Sciences Beijing 100190 China; ^2^ School of Chemistry and Chemical Engineering Henan Normal University Xinxiang 453007 China; ^3^ Institute of Chemistry Chinese Academy of Sciences Beijing 100190 China; ^4^ State Key Laboratory of Heavy Oil Processing China University of Petroleum Beijing 102249 China

**Keywords:** CO_2_ electroreduction, ionic liquids, single‐atom catalyst, scale‐up, techno‐economic assessment

## Abstract

Accelerating the commercialization of CO_2_ electroreduction is essential for carbon utilization, yet it faces challenges of precious metal catalysts cost and scaling‐up of the corresponding devices. In this study, a low‐cost and tri‐coordinated single‐atom catalyst (SAC) with Ni‐N3 center is fabricated in gram‐scale using metal ionic liquids as precursor. The gram‐scale Ni‐N3 SAC (g‐NiN3) achieves efficient electroreduction of CO_2_ to CO (eCO_2_‐to‐CO) with a maximum Faradaic efficiency of 98.9% at 2.8 V in a 2 × 2 cm^2^ membrane electrode assembly (MEA) cell, and CO selectivity exceeds 90% during 100 h electrolysis at 100 mA·cm^−2^. Moreover, the g‐NiN3 is tested in a scale‐up MEA reactor (10 × 10 cm^2^), which can not only show 97.1% CO Faradaic efficiency with a reaction current of 6.07 A but also achieves a CO_2_ single‐pass conversion of 41.0%, corresponding to energy efficiency of the system as high as 43.1%. The overall performance of g‐NiN3 is one of the state‐of‐the‐art systems for eCO_2_‐to‐CO. In addition, the scale‐up device stably generates CO at a high rate of 12.0 L·kW·h^−1^ over continuous CO_2_ electrolysis. The techno‐economic assessment demonstrates that the eCO_2_‐to‐CO using g‐NiN3 can realize CO production cost of 1.08 $·kg^−1^, and shows great profitability prospects in the future.

## Graphical abstract

A low‐cost and tri‐coordinated SAC (g‐NiN3) is prepared in gram‐scale using metal ionic liquid [Bmim]_2_[NiCl_4_] as precursors. The g‐NiN3 achieves an excellent overall performance for eCO_2_‐to‐CO in a large‐scale MEA reactor. The overall performance in g‐NiN3 is superior to that of commercial Ag nanopowders, and manifests as one of the state‐of‐the‐art systems for eCO_2_‐to‐CO.

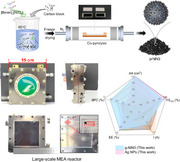

## 1 Introduction

The electroreduction of CO_2_ combined with green electricity provides a sustainable way to produce high value‐added chemicals or fuels.^[^ ^1^, ^2^ ^]^ Among the major products of CO_2_ electroreduction, gaseous CO is one of the most promising products with high economic returns, not only due to high product selectivity but also because of easy separation in liquid electrolyte, which can be further combined with tandem reactors to synthesize multi‐carbon products.^[^ ^3^, ^4^ ^]^ However, the low CO_2_ conversion efficiency makes it difficult to achieve profitability. The single‐pass conversion (SPC) of CO_2_ is closely related to the costs of CO_2_ electroreduction feedstock and product separation.^[^ ^5^ ^]^ A higher SPC means more CO_2_ can be converted, which not only improves product yield but also reduces product separation costs, leading to lower production costs per unit. Taking the electroreduction of CO_2_ to CO (eCO_2_‐to‐CO) as an example, when the CO_2_ SPC was ≈10%, the separation cost of gas products through pressure swing adsorption (PSA) accounted for ≈23% of the total cost. However, the separation cost would decreased to 6% when the CO_2_ SPC reached 50%.^[^ ^6^ ^]^ In alkaline or neutral electrolytes, each generation of CO will produce two OH^−^ ions and thus consume additional CO_2_ to form carbonates. Therefore, the theoretical SPC of CO_2_ is hard to exceed 50% under alkaline conditions.^[^ ^7^ ^]^ Although the above issues can be addressed in acidic electrolytes, it is often accompanied by severe hydrogen evolution reaction (HER) and low CO selectivity.^[^ ^8^, ^9^ ^]^ Recently, Sargent et al and others reported that over 50% SPC of CO_2_ under acidic conditions can be achieved, marking a significant breakthrough in this field.^[^ ^10^ ^]^ However, the satisfactory SPC of CO_2_ could only be achieved at very low CO_2_ flow rates below 10 mL min^−1^,^[^ ^11^, ^12^, ^13^ ^]^ which is not suitable for practical industrial applications. As a result, how to simultaneously maintain a superior CO selectivity and an excellent CO_2_ SPC at high CO_2_ flow rates is a great challenge for realizing the commercialization of eCO_2_‐to‐CO technology.

In addition, the currently recognized catalytic materials with superior eCO_2_‐to‐CO performance are precious metals such as gold and silver, which severely limit their future applications due to their high‐cost inputs.^[^ ^14^, ^15^ ^]^ Atomically dispersed metal (M)‐N sites embedded into carbon‐based materials (M‐N‐C) have provided new opportunities for eCO_2_‐to‐CO conversion due to their high atomic utilization efficiency, low‐cost metal centers, unique electronic structure, and outstanding Faradaic efficiency (FE) of CO.^[^ ^16^, ^17^, ^18^ ^]^ In addition, atomically dispersed M‐N sites also show great potential in energy conversion and storage, such as Zn‐CO_2_ batteries.^[^ ^19^, ^20^, ^21^ ^]^ However, the majority of single‐atom catalysts (SACs) applied in eCO_2_‐to‐CO conversion are based on tetra‐coordinated active centers of M‐N4.^[^ ^22^, ^23^ ^]^ The latest studies indicated that the direct synthesis of SACs with M‐N3 sites by breaking the planar symmetry of the M‐N4 centers is able to maximize the catalytic activity for eCO_2_‐to‐CO.^[^ ^24^, ^25^, ^26^ ^]^ Therefore, orienting synthesis of SACs with tri‐coordinated M‐N3 centers for eCO_2_‐to‐CO becomes more attractive. However, the current synthesis scale of SACs is almost only at the milligram level,^[^ ^27^, ^28^ ^]^ which makes it difficult to match the consumption of large‐scale applications in future. Scalable production of SACs to at least the gram‐scale synthesis remains a challenge in this field. In addition, although the reported M‐N‐C SACs have demonstrated excellent CO selectivity in H‐type cell or flow cell, their comprehensive performance (FE_CO_, CO_2_ SPC, energy efficiency (EE), stability, etc.) for CO_2_ electrolysis in membrane electrode assembly (MEA), which are easier to scale up, still needs to be further investigated.^[^ ^29^, ^30^ ^]^ In particular, it is urgent to evaluate the performance of eCO_2_‐to‐CO in large‐scale reactors to assess their feasibility for industrial applications.

With this in mind, we are committed to realizing the gram‐scale synthesis of SACs combined with the large‐scale MEA reactors to achieve a breakthrough in the overall performance of eCO_2_‐to‐CO. Herein, we used metal ionic liquid of 1‐butyl‐3‐methylimidazolium nickel tetrachloride ([Bmim]_2_[NiCl_4_]) as precursors to achieve the gram‐scale synthesis of Ni SACs with tri‐coordinated Ni‐N3 sites via a simple two‐step method. The gram‐scale Ni‐N3 (g‐NiN3) not only boasts a lower cost compared to commercial Ag nanopowders (Ag NPs), but also delivers outstanding performance and durability for eCO_2_‐to‐CO. Moreover, an eCO_2_‐to‐CO scale‐up device equipped with a 10 × 10 cm^2^ large‐scale MEA reactor was designed and established. In this scale‐up device, the overall performance of g‐NiN3, including FE_CO_, reaction current, CO_2_ SPC, EE, and stability was systematically evaluated, and the techno‐economic assessment (TEA) was analyzed the industrial application potentials of eCO_2_‐to‐CO technology combing MEA cell and novel g‐NiN3 SACs.

## 2 Results and Discussion

**Figure 1:**
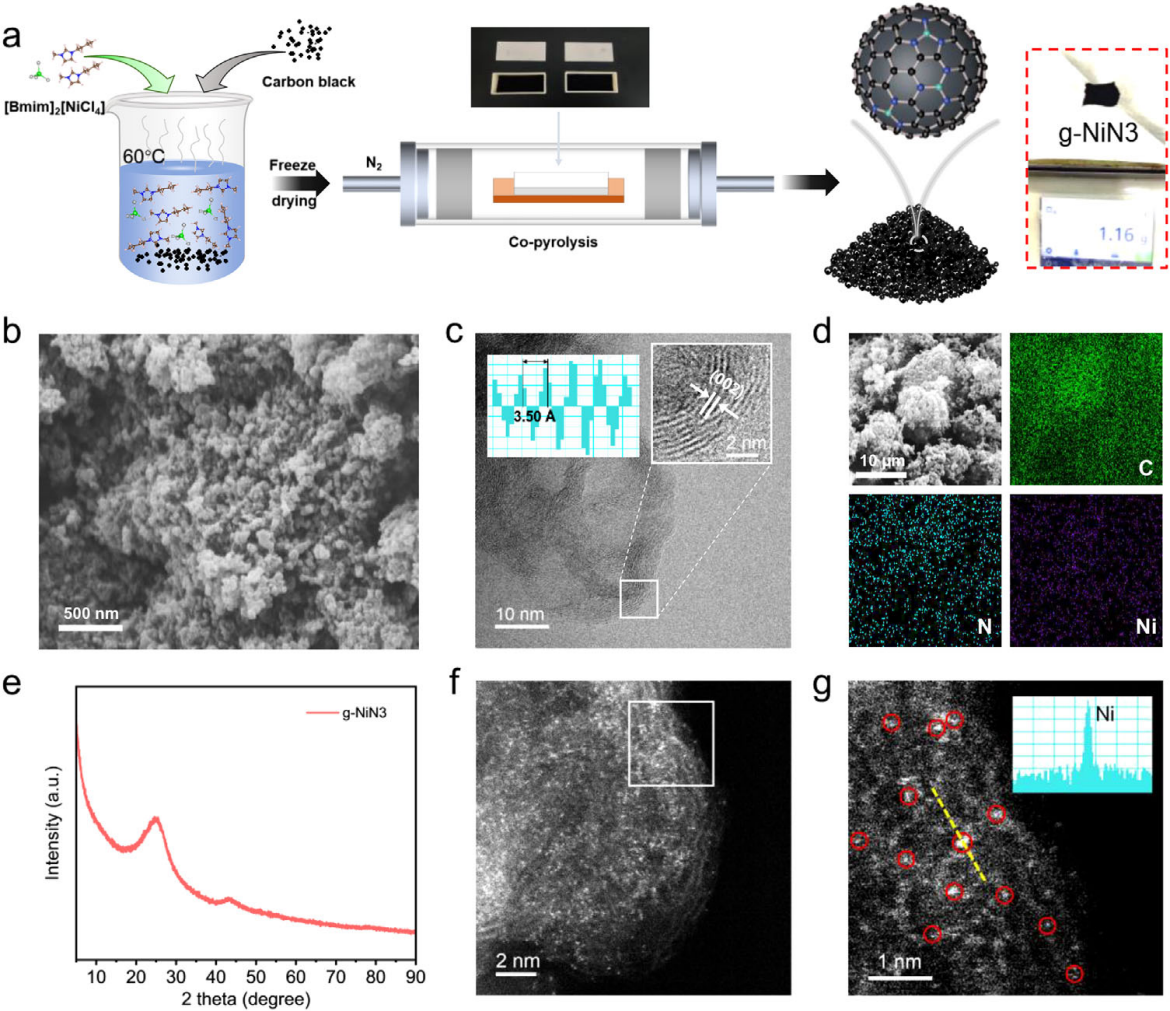
a) Synthesis procedure of g‐NiN3. b) SEM images of g‐NiN3. c) TEM images of g‐NiN3. d) EDS mappings of g‐NiN3. e) XRD results of g‐NiN3. f) AC‐HAADF‐STEM image of g‐NiN3 with numerous bright spots of atomically dispersed Ni; g) Ni intensity signal of g‐NiN3 and partially marked with a red circle.

The synthesis procedure of g‐NiN3 is shown in **Figure** **1**a. The g‐NiN3 could be obtained by adopting metal ionic liquid [Bmim]_2_[NiCl_4_] as both N source and Ni source doped with carbon black after a two‐step method, and the detailed synthesis procedure was described in the Supporting Information. The scanning electron microscopy (SEM) images showed that the morphology of g‐NiN3 is a coral‐like porous structure (Figure 1b). The surface area of the porous g‐NiN3 is 450.547 m^2^ g^−1^, and the higher ECSA value of 123.15 mF cm^−2^ also confirms that g‐NiN3 has rich active sites on the surface (Figure S1, Supporting Information), which can facilitate CO_2_ mass transfer on the surface of the catalyst, thereby promoting eCO_2_‐to‐CO. Moreover, only a crystal spacing of 3.5 Å attributed to graphitic carbon (002) facets could be observed in the transmission electron microscopy (TEM) images (Figure 1c), indicating the absence of any metallic phases of Ni species in g‐NiN3. The energy‐dispersive X‐ray spectroscopy (EDS) mapping in Figure 1d further showed that the Ni and N species are successfully introduced into the carbon matrix and are uniformly distributed in g‐NiN3. Diffraction peaks attributed to graphitic carbon were only observed at 24.6° and 43.4° in the X‐ray diffraction (XRD) results without any other diffraction signals (Figure 1e), suggesting that the Ni species in g‐NiN3 exist as single atoms or clusters. The atomic level image of g‐NiN3 was observed by aberration‐corrected high‐angle annular darkfield scanning transmission electron microscopy (AC‐HAADF‐STEM), and numerous bright spots were uniformly dispersed on the surface of the catalyst in Figure 1f. The Ni intensity signal further revealed the atomic level dispersion of Ni species in g‐NiN3, which preliminarily confirmed that the g‐NiN3 is a single‐atom catalyst (SAC). The Ni content in g‐NiN3 was 2.3 wt.% as tested by inductively coupled plasma‐atomic emission spectrometry (ICP‐AES) as shown in Figure S2 (Supporting Information).

**Figure 2:**
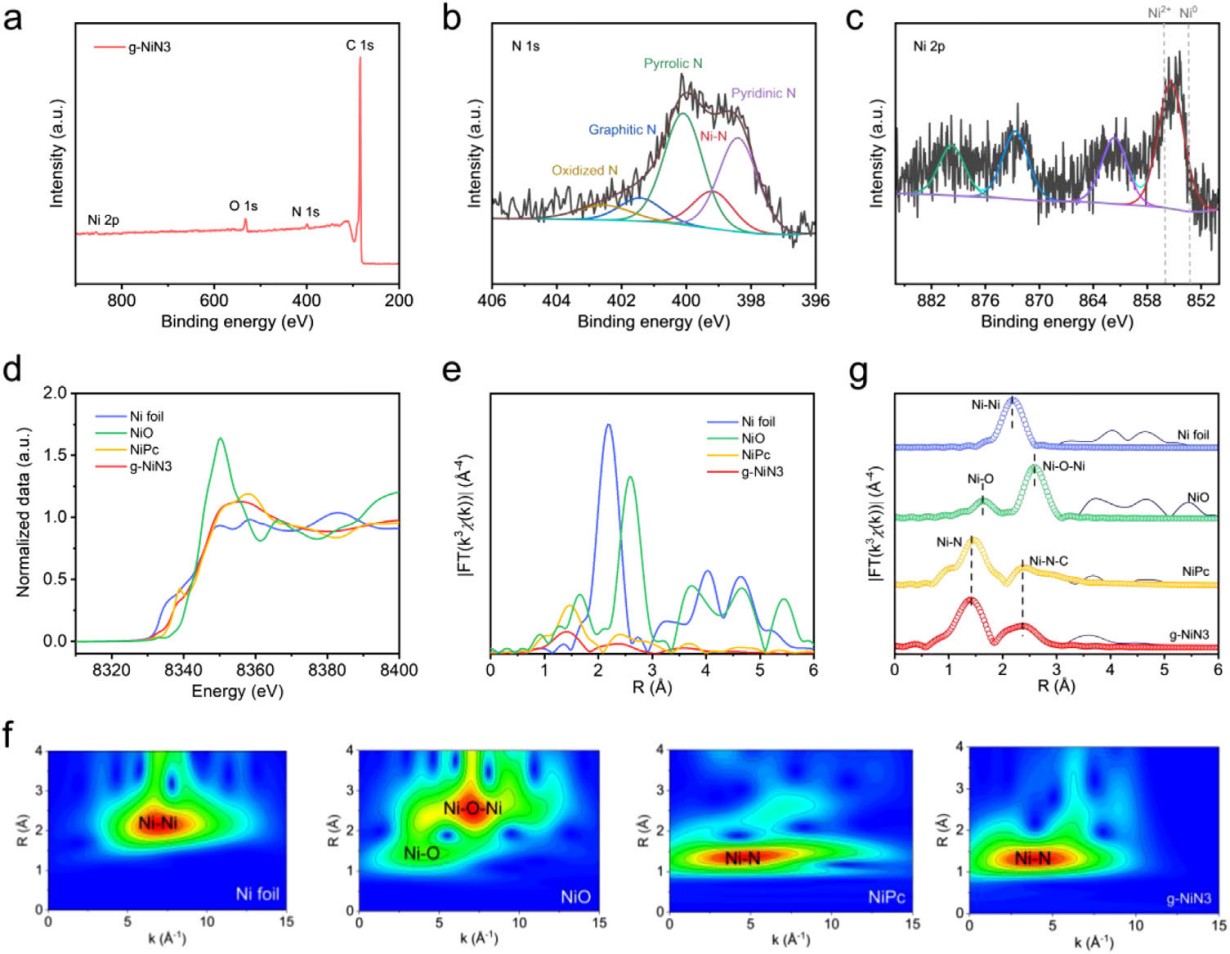
a) XPS survey results of g‐NiN3. b,c) High‐resolution N 1s and Ni 2p spectrum of g‐NiN3. d) Ni K‐edge EXANE and e) EXAFS results of g‐NiN3. f) Wavelet transformation results of g‐NiN3. g) Ni K‐edge fitting curves of g‐NiN3 in R space.

To further analyze the fine structures of g‐NiN3, X‐ray photoelectron spectroscopy (XPS) was first conducted to determine the chemical compositions and elemental chemical valence. As shown in the XPS results of **Figure** **2**a, there were clear peak signal responses of Ni and N elements, which suggests that [Bmim]_2_[NiCl_4_] acts as a precursor to successfully introduce Ni and N species into the carbon matrix. Among them, the high‐resolution N 1s spectrum of g‐NiN3 could be split into oxidized N (402.6 eV), graphite N (401.5 eV), pyrrolic N (400.2 eV), and pyridinic N (398.6 eV),^[^ ^31^, ^32^ ^]^ respectively (Figure 2b). In addition, a characteristic new peak at 399.2 eV attributed to the Ni–N signal was detected,^[^ ^33^ ^]^ indicating the bonding between Ni and N atoms in g‐NiN3. The binding energy of Ni 2p_3/2_ was 855.3 eV in the high‐resolution Ni 2p spectrum (Figure 2c), which is located between 853.5 eV of Ni (0) and 856.0 eV of Ni (II), indicating that the valence state of Ni atoms is between 0 and +2 in g‐NiN3.

X‐ray absorption spectroscopy (XAS) was further performed for in‐depth analysis of the coordination structure of Ni atoms in g‐NiN3. The Ni K‐edge X‐ray absorption near‐edge structure (XANES) results showed that the pre‐edge energy of Ni is between that of Ni foil and NiO (Figure 2d), which further confirms a 0–+2 valence state of Ni atoms in g‐NiN3. The results of Ni K‐edge extended X‐ray absorption fine structure (EXAFS), obtained by Fourier transform processing, are shown in Figure 2e. The Ni─Ni bond in Ni foil, and the Ni─O and Ni─O─Ni bonds in NiO were visible at 2.17, 1.60, and 2.55 Å, respectively, but almost disappeared in g‐NiN3. However, the main peak was located ≈1.41 Å in g‐NiN3, which corresponds to the Ni–N scattering in nickel phthalocyanine (NiPc). The above analysis results indicated that the Ni species in g‐NiN3 exists in the form of Ni─N bonds, which mimics the Ni─N configuration in NiPc and directly confirms the successful synthesis of gram‐scale Ni SACs. To further analyze the coordination environment of Ni atoms, the EXAFS wavelet transformation was performed on backscattered atoms in R‐space by providing radial distance resolution and k‐space resolution. In Figure 2f, the maximum intensity at 4.1 Å^−1^ ascribed to the Ni─N bonds showed in the wavelet transformation contour plots of g‐NiN3, which only corresponded to the maximum intensity of Ni─N bonds in NiPc, and no other forms of Ni atoms bonding. The wavelet transformation analysis further demonstrated that the Ni atom bonds with the N atom in an atomically dispersed form and anchored to the carbon matrix. According to the quantitative fitting results of EXAFS (Figure 2g; Table S1, Supporting Information), the fitting coordination number of the Ni‐N path was 3.1 ± 0.2, which means that the Ni atom is bonded with threefold N atoms to form a central structure of Ni‐N3 in g‐NiN3 (Figure S3, Supporting Information). As a result, the gram‐scale synthesis of Ni‐based SACs was successfully achieved using metal ionic liquid [Bmim]_2_[NiCl_4_] as both Ni and N sources, and the quantitative analysis and characterization confirmed that the g‐NiN3 is a single‐atom structure with tri‐coordinated Ni‐N3 centers. According to our previous work,^[^ ^34^ ^]^ SACs with tri‐coordinated centers exhibit superior CO_2_‐to‐CO performance compared to symmetric tetra‐coordinated SACs, and the method using metal ionic liquid precursors for the synthesis of SACs with tri‐coordinated centers was not only demonstrated good universality but has also proven to exhibit excellent scalability of g‐NiN3 production in this work.

**Figure 3:**
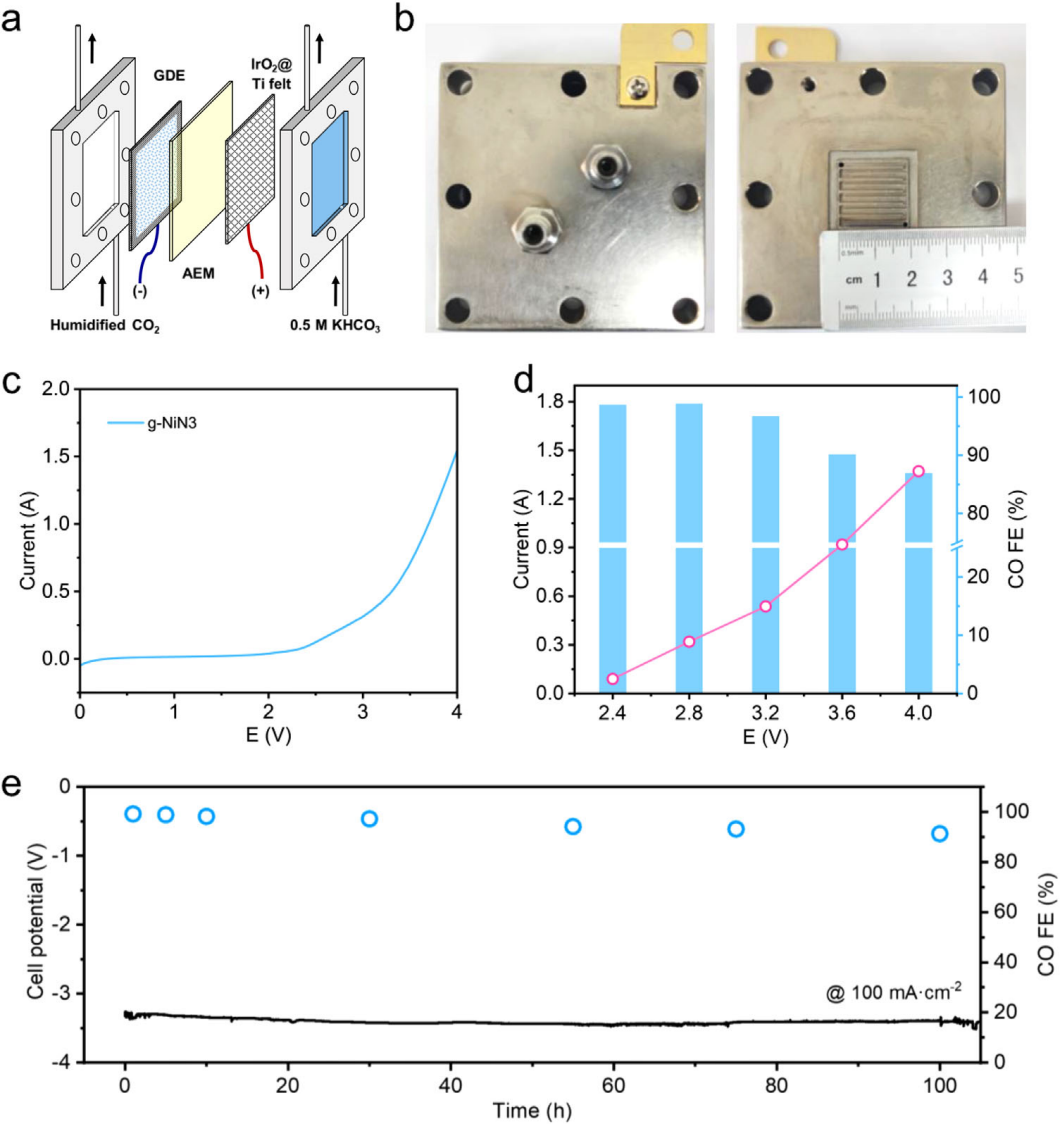
a) Configuration structure of the MEA cell. b) Images of the bipolar plates of MEA cell with an electrode active area of 2 × 2 cm^2^. c) LSV result of g‐NiN3 in a MEA cell. d) The eCO_2_‐to‐CO performance of g‐NiN3 under different potentials. e) A continuous 100 h CO_2_ electrolysis at a constant current density of 100 mA·cm^−2^.

Considering the poor CO_2_ mass transfer in H‐type cell and the electrolyte flooding in flow cell, an MEA electrolysis system, as shown in Figure S4 (Supporting Information) was constructed to evaluate the eCO_2_‐to‐CO performance of g‐NiN3. The core MEA cell structure was configured as shown in **Figure** **3**a, in which the cathode, anode, and anion exchange membrane are directly compressed together to form a zero‐gap module to facilitate CO_2_ electroreduction. The bipolar plates of the MEA cell were depicted in Figure 3b, and both the cathode and anode plates adopted the flow pattern of a single serpentine channel with an electrode active area of 2 × 2 cm^2^. The linear sweep voltammetry (LSV) test was first conducted in this system and the result was shown in Figure 3c. It was evident that the current of CO_2_ electroreduction reaction rapidly increases when the applied potential exceeds 2.0 V. When the applied potential is 3.6 V, the total reaction current of the system reaches 1.0 A, indicating that the g‐NiN3 exhibits high CO_2_ electroreduction activity in the MEA cell. Subsequently, the eCO_2_‐to‐CO performance under different potentials was further performed in the MEA cell. The test results in Figure 3d demonstrated that the g‐NiN3 can achieve a CO FE greater than 90% under a wide potential range from 2.4 to 3.6 V. Specifically, it can reach a CO FE of 98.9% and a current density of 80 mA cm^−2^ at a lower potential of 2.8 V. Even as the potential increases to 4.0 V, the system can still maintain an 86.9% CO selectivity with a total reaction current of 1.4 A (equivalent to a current density of 350 mA·cm^−2^), demonstrating excellent performance of eCO_2_‐to‐CO.

The long‐term stability of catalytic materials is one of the key factors in evaluating whether CO_2_ electroreduction technology can realize the applications. Therefore, the durable test of g‐NiN3 was further conducted using the MEA electrolysis system at a constant current density of 100 mA·cm^−2^, and the results are shown in Figure 3e. After the continuous 100 h CO_2_ electrolysis, the FE of eCO_2_‐to‐CO was still 91.2%, and the CO FEs were all maintained at above 90% with ignorable changes in the cell potential during the durable CO_2_ electrolysis. The TEM image of g‐NiN3 after electrolysis further confirmed that no noticeable changes are observed in the materials morphology (Figure S5, Supporting Information), and only peaks ascribed to the facets of graphitic carbon can be detected in the XRD results by comparing with the results of blank carbon paper. The high‐resolution N 1s spectrum of g‐NiN3 after CO_2_ electrolysis showed that the characteristic Ni–N peak at 399.2 eV still exists, and the Ni 2p_3/2_ peak at 855.4 eV in the high‐resolution Ni 2p spectrum is still within the range between Ni (0) at 853.5 eV and Ni (II) at 856.0 eV, indicating that the Ni single atoms are not reduced to the Ni species with a 0‐valence state. In addition, the HAADF‐STEM results showed that there are still a large number of single bright spots scattered in the image, and most of the Ni species in the element mapping show a single dispersed state. The above results indicated that the g‐NiN3 exhibits excellent electrochemical durability after long‐cycle electrolysis without any Ni atom reduction or metal phase formation.

**Figure 4:**
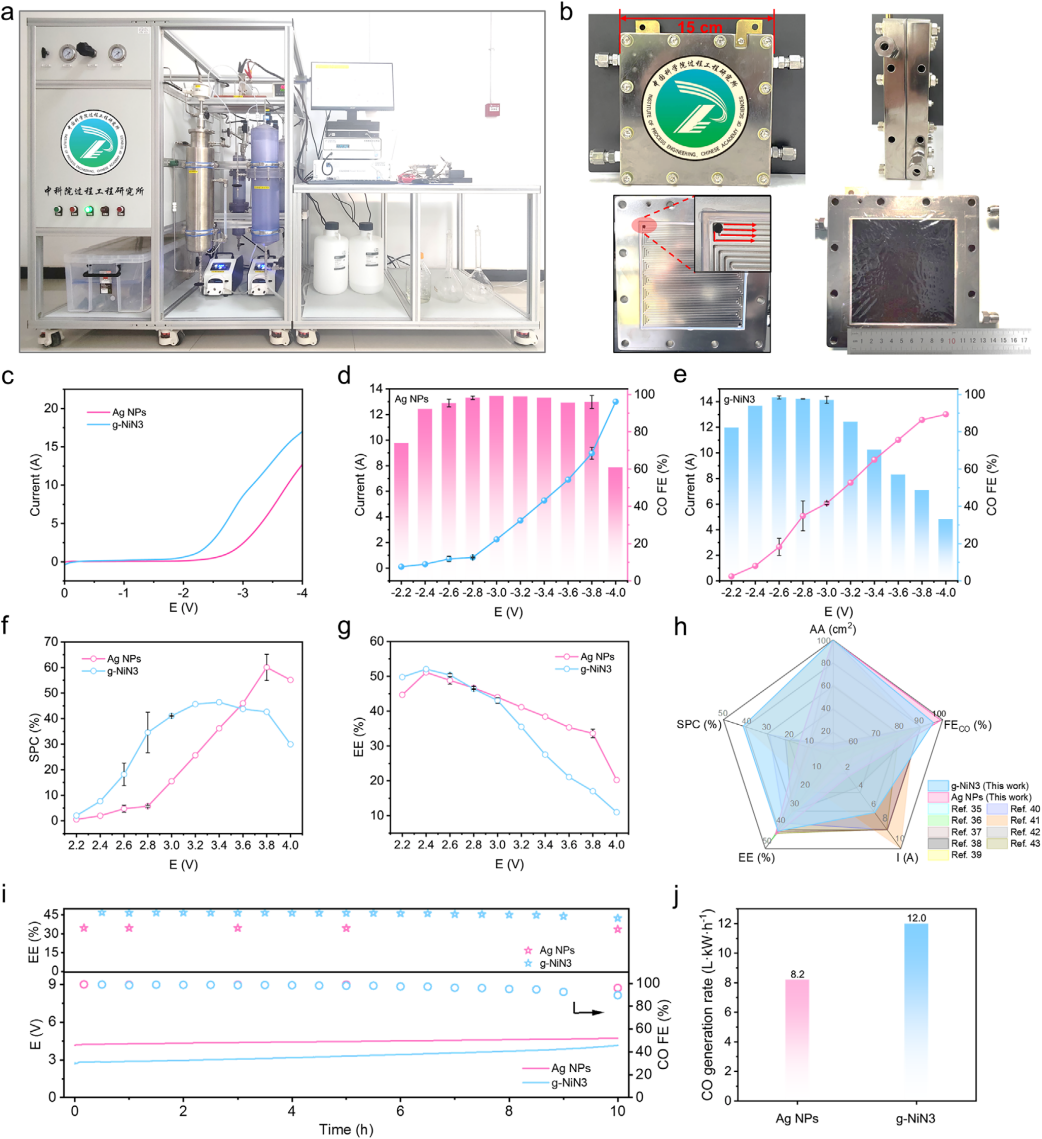
a) Image of the CO_2_ electroreduction scale‐up device. b) Images of the large‐scale MEA reactor with an electrode area of 10 × 10 cm^2^. c) LSV results of g‐NiN3 and Ag NPs in the large‐scale MEA reactor. The eCO_2_‐to‐CO performance of d) g‐NiN3 and e) Ag NPs under different potentials. f) SPCs and g) EEs of the eCO_2_‐to‐CO system in g‐NiN3 and Ag NPs. h) Comparison of overall performance with state‐of‐the‐art eCO_2_‐to‐CO performance.^[^ ^35^, ^36^, ^37^, ^38^, ^39^, ^40^, ^41^, ^42^, ^43^ ^]^ i) Stability evaluation of CO_2_ electrolysis in the scale‐up device for 10 h, with Ag NPs and g‐NiN3 conducted in a constant current of 10 and 6 A, respectively. j) Comparison of CO generation rates between g‐NiN3 and Ag NPs.

The practical industrial feasibility of CO_2_ electroreduction depends on the scaling‐up studies of this technology. A CO_2_ electroreduction scale‐up device, as depicted in **Figure** **4**a based on the process diagram in Figure S6 (Supporting Information), was constructed with a detailed configuration described in the Supporting Information. The core component of this device is a large MEA reactor as shown in Figure 4b, with an electrode active area of 10 × 10 cm^2^, 25 times larger than the MEA cell described above. The LSV results of g‐NiN3 exhibited a higher reaction current with a lower onset potential ≈2.0 V in Figure 4c, suggesting superior catalytic activity for eCO_2_‐to‐CO compared to that of Ag NPs. The Ag NPs (Alfa Aesar, 99.9 wt.%, 20–40 nm) were used directly without further treatment. Subsequently, the eCO_2_‐to‐CO performance of g‐NiN3 was further investigated under different potentials in this scale‐up device and compared with that of the commercial Ag NPs, which are currently recognized as excellent candidates due to great selectivity for eCO_2_‐to‐CO. As shown in Figure 4d,e, the eCO_2_‐to‐CO performance in Ag NPs could achieve a CO FE exceeding 90.0% over a wide potential range of 2.4–3.8 V, with the highest CO FE of 99.2% at 3.0 V, corresponding to a total reaction current of 2.24 A, with a CO partial current of only 2.22 A (22.2 mA·cm^−2^). Conversely, the g‐NiN3 in this work could show a high CO FE of 97.7% and a total reaction current of 5.08 A, corresponding to a CO partial current of 4.96 A (49.6 mA cm^−2^), which surpass Ag NPs at 2.8 V. When the potential was increased to 3.0 V, the g‐NiN3 still maintained a CO FE of 97.1% with a CO partial current as high as 5.89 A (58.9 mA·cm^−2^), ≈2.65 times that of Ag NPs under the same potential conditions in this device. Hence, both g‐NiN3 and Ag NPs can sustain superior CO selectivity at 3.0 V, while g‐NiN3 synthesized in this work shows almost a twofold increase in catalytic activity for eCO_2_‐to‐CO compared to Ag NPs.

From the viewpoint of industrial applications, the EE and SPC of g‐NiN3 and Ag NPs were analyzed for eCO_2_‐to‐CO at different potentials to evaluate their application potentials, respectively. The CO_2_ flow rate of this scale‐up device was set at 100 mL·min^−1^ for all the tests. The SPC of CO_2_ is usually positively correlated with the effective current of the system to generate the target product. According to the SPC results shown in Figure 4f, when the applied potential was over 3.6 V, the more severe HER in g‐NiN3 resulted in lower CO partial currents compared to Ag NPs, causing a decrease in its SPC values lower than those of Ag NPs. Specifically, the eCO_2_‐to‐CO in Ag NPs exhibited a peak SPC of 60.0% at 3.8 V. However, the CO partial currents in g‐NiN3 system are superior to those of Ag NPs at potentials below 3.6 V, meaning a faster CO_2_ molecule consumption rate. Thus, the SPC values for eCO_2_‐to‐CO in g‐NiN3 are higher than those of Ag NPs in the potential range below 3.6 V, in which the peak SPC was up to 46.4% at 3.4 V. In addition, it was concluded from Figure 4g that the EEs of eCO_2_‐to‐CO in g‐NiN3 are all superior to those of Ag NPs below 2.8 V. At 2.4 V, the EE of the eCO_2_‐to‐CO system in g‐NiN3 reached the highest value of 52.1%, which is one of the most outstanding systems in eCO_2_‐to‐CO. However, the increasingly severe HER in g‐NiN3 causes its EE to start lagging behind that of Ag NPs and gradually decline at higher potentials. In summary, g‐NiN3 not only exhibited over 97.1% FE_CO_ and a reaction current of 6.07 A in the largest single‐MEA reactor, but also achieved an SPC of 41.0% with a corresponding EE of 43.1% at 3.0 V, which realizes the synergistic optimization of multiple parameters, and is one of the state‐of‐the‐art reaction systems of eCO_2_‐to‐CO overall performance (Figure 4h; Table S2, Supporting Information).^[^ ^35^, ^36^, ^37^, ^38^, ^39^, ^40^, ^41^, ^42^, ^43^ ^]^

Finally, the scale‐up device was used to evaluate the continuous eCO_2_‐to‐CO performance of Ag NPs and g‐NiN3 under constant current conditions of 10 and 6 A, respectively, and the results are shown in Figure 4i. During the continuous 10 h electrolysis, there was no significant fluctuation in the cell potential of either the Ag NPs or g‐NiN3 system. Moreover, the CO FEs were both consistently above 90% after 10 h of continuous CO_2_ electrolysis. Notably, eCO_2_‐to‐CO in g‐NiN3 was able to achieve CO selectivity comparable to that of Ag NPs at a lower potential, resulting in a higher EE of 45.0% compared to only 34.7% for Ag NPs. According to the total CO generation from the CO_2_ electroreduction in Ag NPs and g‐NiN3 for 10 h, as shown in Figure S7 (Supporting Information), the Ag NPs and g‐NiN3 system generates a total of 41.0 and 24.0 L of CO, respectively. The total electricity consumption for the Ag NPs system was 0.5 kW h, which corresponds to a CO generation rate of 8.2 L kW·h^−1^ during the continuous 10 h CO_2_ electrolysis. However, as illustrated in Figure 4j, a higher CO generation rate of 12.0 L·kW·h^−1^ was obtained in g‐NiN3 due to its greater EEs than the Ag NPs system. Furthermore, the CO generation in both Ag NPs and g‐NiN3 showed steady growth, indicating excellent stability in the scale‐up device. It is worth noting that the scalable synthesis of g‐NiN3 in this work exhibits the advantages of low‐cost and simple synthesis method compared to Ag NPs, and demonstrates a broad application potential by achieving comparable CO selectivity and CO_2_ electrolysis stability to Ag NPs under higher EE conditions.

**Figure 5:**
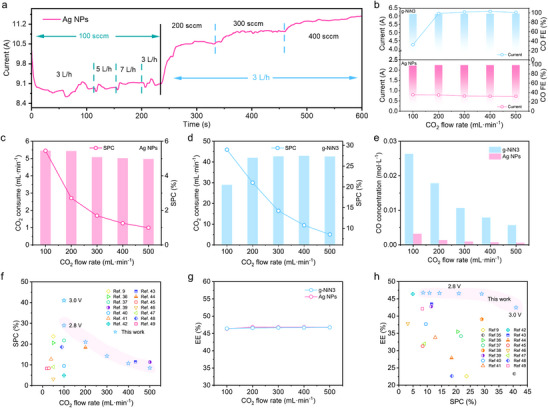
a) Sensitivity analysis of electrolyte and CO_2_ flow rate in the scale‐up device using Ag NPs at 3.8 V. b) The effect of different CO_2_ flow rates on eCO_2_‐to‐CO performance in g‐NiN3 and Ag NPs. CO_2_ single‐pass consumption and SPC in c) Ag NPs and d) g‐NiN3 under different CO_2_ flow rates. e) CO concentration in products under different CO_2_ flow rates. f) Comparison of CO_2_ SPC of g‐NiN3 with other eCO_2_‐to‐CO systems.^[^ ^9^, ^36^, ^37^, ^39^, ^40^, ^41^, ^42^, ^43^, ^44^, ^45^, ^46^, ^47^, ^48^, ^49^ ^]^ (d) EEs of eCO_2_‐to‐CO system under different CO_2_ flow rate, all results of Ag NPs and g‐NiN3 were both conducted at 2.8 V. h) Plots of comparison of the EEs against SPCs in g‐NiN3 with other eCO_2_‐to‐CO systems.^[^ ^9^, ^35^, ^36^, ^37^, ^38^, ^39^, ^40^, ^41^, ^42^, ^43^, ^44^, ^45^, ^46^, ^47^, ^48^, ^49^ ^]^

Changes in operation conditions may affect the eCO_2_‐to‐CO performance in the scale‐up device, sensitivity analyses including the anode electrolyte flow rate and cathode CO_2_ flow rate in the MEA reactor were performed at 3.8 V using Ag NPs, and the results were shown in **Figure** **5**a. During the first 230 s of electrolysis with a constant CO_2_ flow rate of 100 mL·min^−1^, when the KHCO_3_ electrolyte flow rate was increased from 3 to 5 L·h^−1^ or 7 L·h^−1^, the system reaction current was maintained at ≈9.1 A, with almost no significant change, indicating a minor impact of the anode electrolyte flow rate variation on the eCO_2_‐to‐CO performance. Subsequently, an increase in the cathode CO_2_ flow rate from 100 to 200 mL·min^−1^ led to a noticeable increase in the overall reaction current from 9.1 to 10.5 A when the anode electrolyte flow rate was kept constant at 3 L·h^−1^. Further increases in the CO_2_ flow rate from 200 to 300 mL·min^−1^, and from 300 to 400 mL·min^−1^ resulted in a slight change of reaction currents. Sudden changes or increases in reaction current indicated an impact on the CO_2_ electroreduction system. As a result, the changes in the CO_2_ flow rate have a greater impact on the CO_2_ electroreduction compared to in the effect of the anode KHCO_3_ electrolyte flow rate.

Based on the above results, the effect of different CO_2_ flow rates on eCO_2_‐to‐CO performance was further investigated in the scale‐up device. The eCO_2_‐to‐CO performance under different CO_2_ flow rates for g‐NiN3 and Ag NPs was tested at 2.8 V, with a range of CO_2_ flow rates from 100 to 500 mL·min^−1^ in increments of 100 mL·min^−1^, and the results were shown in Figure 5b. Regardless of whether CO_2_ electroreduction was carried out in Ag NPs or g‐NiN3, the CO selectivity was hardly affected by different CO_2_ flow rates. For instance, the CO FE remained at 98.0% as the CO_2_ flow rate increased from 100 to 500 mL·min^−1^ during the CO_2_ electroreduction at 2.8 V using g‐NiN3. However, a significant increase in the reaction current for CO_2_ electroreduction was observed in g‐NiN3 with an increase in CO_2_ flow rate from 100 to 200 mL·min^−1^. In contrast, due to the low CO_2_ reaction rate in Ag NPs with a current of only 0.8 A (8.0 mA·cm^−2^) for CO_2_ electroreduction at 2.8 V, the effect of the reaction current on this system changes insignificantly when the CO_2_ flow rate is varied. Nevertheless, a sudden change in reaction current can also be observed in Ag NPs at 3.8 V when the CO_2_ flow rate increases from 100 to 200 mL·min^−1^ (Figure S8, Supporting Information). Thus, under higher reaction current conditions, the reaction rate of eCO_2_‐to‐CO further increases with the rise of CO_2_ flow rate. However, when the CO_2_ flow rate is further increased from 200 to 500 mL·min^−1^, there is no significant increase in the reaction current for either the Ag NPs or g‐NiN3 systems, which may be constrained by the capacity of the large‐scale MEA reactor.

In Figure 5c,d, the consumption of CO_2_ in g‐NiN3 significantly increased from 29.0 to 42.0 mL·min^−1^ as the CO_2_ flow rate rose from 100 to 200 mL·min^−1^, whereas the CO_2_ consumption in Ag NPs only changed slightly from 5.5 to 5.4 mL·min^−1^. With further increase in the CO_2_ flow rate, the consumption of CO_2_ remains relatively stable in both Ag NPs and g‐NiN3, which is also consistent with the fact that the reaction current of the g‐NiN3 system increased significantly when the CO_2_ flow rate was increased from 100 to 200 mL·min^−1^, but the change was not obvious in Ag NPs. The escalation in the consumption of CO_2_ indicates a more intense reaction rate, manifested by a significant increase in the reaction current, and further raising the CO_2_ flow rate results in a nearly negligible change in the CO_2_ consumption, corresponding to almost no further variation in the reaction current of the CO_2_ electroreduction system. When the CO_2_ flow rate is 100 mL·min^−1^, the SPC of CO_2_ electroreduction in Ag NPs at 2.8 V was only 5.4%, while the SPC of CO_2_ in g‐NiN3 was as high as 29.0%. As the CO_2_ flow rate gradually increases, the SPC of CO_2_ begins to gradually decrease due to the limited SPC of CO_2_ in the scale‐up device. When the CO_2_ flow rate was 500 mL·min^−1^, the SPC of CO_2_ in Ag NPs and g‐NiN3 decreased to 1.0% and 8.5%, respectively. As the CO_2_ flow rate increased from 100 to 500 mL·min^−1^, the amount of unreacted CO_2_ increased in the scale‐up device, ultimately leading to a gradual decrease in CO concentration of the products. For instance, CO concentration of the products decreased from 3.2 to 0.6 mmol·L^−1^ in Ag NPs, and that of g‐NiN3 decreased from 26.4 to 5.7 mmol·L^−1^ as shown in Figure 5e. Compared to other electrocatalyst systems shown in Figure 5f and Table S3 (Supporting Information),^[^ ^9^, ^36^, ^37^, ^39^, ^40^, ^41^, ^42^, ^43^, ^44^, ^45^, ^46^, ^47^, ^48^, ^49^ ^]^ the eCO_2_‐to‐CO in g‐NiN3 exhibited excellent SPCs of CO_2_ at different CO_2_ flow rates, especially considering that these results were achieved in a large‐scale MEA reactor.

However, the change in CO_2_ flow rate has an insignificant impact on the EEs of the eCO_2_‐to‐CO system. Moreover, when both g‐NiN3 and Ag NPs were tested at 2.8 V, the CO selectivity remained at 98.0% under different CO_2_ flow rates, and the EEs ≈46.5% of the system were almost the same based on the Equation S3 (Supporting Information) as shown in Figure 5g. In summary, when the flow rate of the CO_2_ electroreduction scale‐up device was maintained at 100 mL·min^−1^ at 2.8 V, the SPC of g‐NiN3 in this work was as high as 29.0%, which was much higher than that of Ag NPs (5.4%) and possessed the comparable EEs with that of Ag NPs. As the CO_2_ flow rate was further increased, the limited capacity of the CO_2_ electroreduction scale‐up device caused a gradual decrease in SPC of the system. Therefore, when the CO_2_ flow rate is 100 mL·min^−1^, the CO_2_ conversion can achieve relatively favorable performance results in the scale‐up device equipped with a 10 × 10 cm^2^ single‐MEA reactor. Notably, the eCO_2_‐to‐CO in g‐NiN3 has achieved record results in the turning between the SPCs of CO_2_ and EEs performance shown in Figure 5h and Table S4 (Supporting Information),^[^ ^9^, ^35^, ^36^, ^37^, ^38^, ^39^, ^40^, ^41^, ^42^, ^43^, ^44^, ^45^, ^46^, ^47^, ^48^, ^49^ ^]^ manifesting as one of the best eCO_2_‐to‐CO systems in a large‐scale MEA reactor.

**Figure 6:**
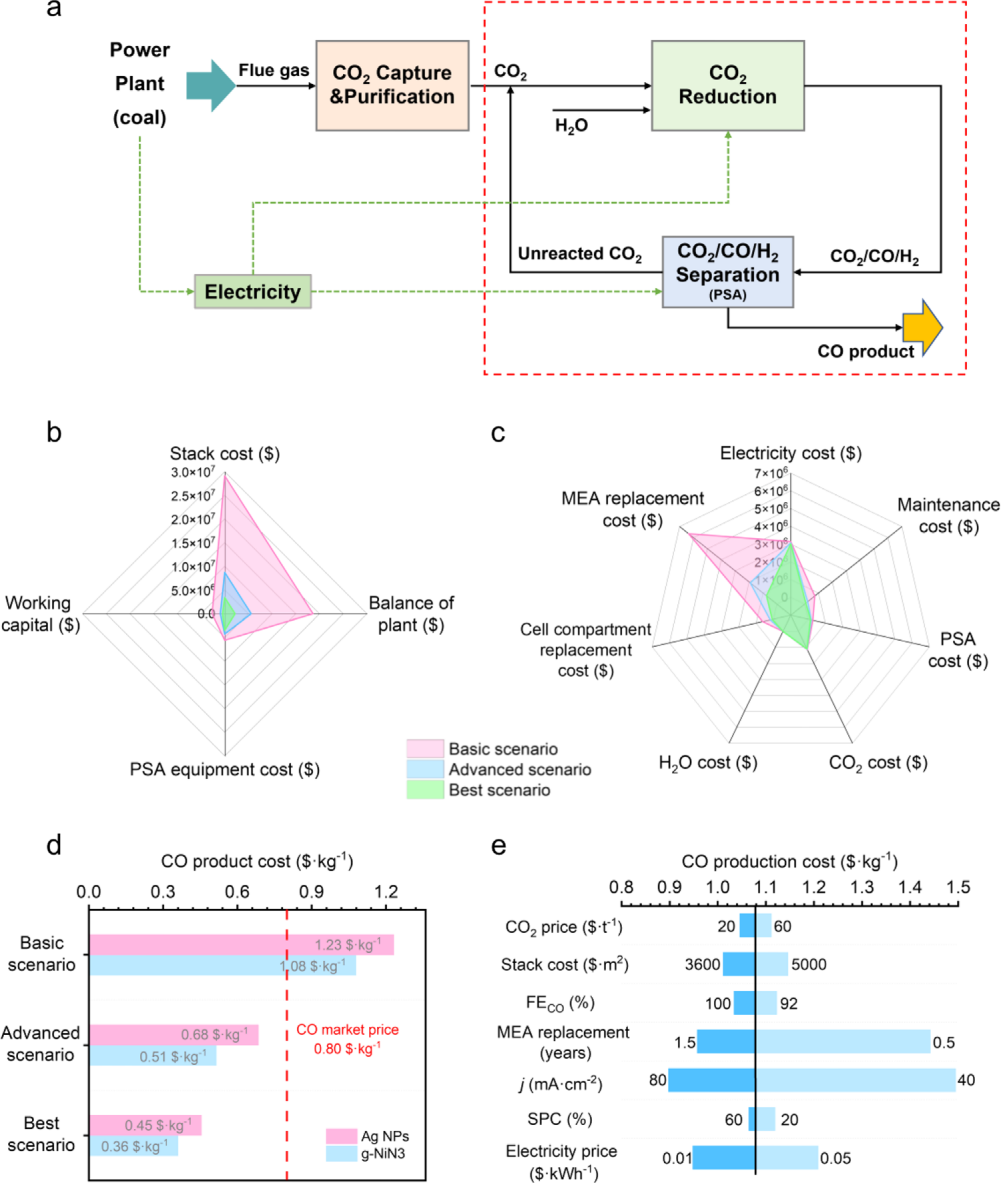
a) TEA model of eCO_2_‐to‐CO. b) The investment costs in different scenarios of CO_2_ electroreduction in g‐NiN3. c) Annual operating costs of in different scenarios of CO_2_ electroreduction in g‐NiN3. d) Comparison of CO production costs for g‐NiN3 and Ag NPs in different scenarios. e) Conditional sensitivity analysis of g‐NiN3 in basic scenarios.

In order to assess the profit potential of CO_2_ electroreduction technology in future applications, a techno‐economic assessment (TEA) of the eCO_2_‐to‐CO was conducted in this study. Taking a typical thermal power plant as an example, flue gas generated by coal combustion is captured and purified into high‐purity CO_2_ by carbon capture technology, which is reduced to CO with H_2_O as the reactant in the electroreduction unit. The high‐purity CO was further separated by the PSA technology, and the unreacted CO_2_ is reintroduced into the electroreduction unit for reaction. The electricity required for the entire process is supplied by the thermal power plant or driven by the green power in the future. The analytical model of TEA was illustrated within the red boundary in **Figure** **6**a, without considering the cost input of the upstream CO_2_ capture and purification processes.

Based on the performance of eCO_2_‐to‐CO by the g‐NiN3 and Ag NPs in this work, it is defined as the basic scenario parameters. Advanced scenarios and best scenarios for eCO_2_‐to‐CO are reasonably set based on the progress of CO_2_ electroreduction technology as shown in Table S5 (Supporting Information), respectively. Constructing a CO_2_ electrochemical plant with a CO production target of 50 t·d^−1^, the TEA of eCO_2_‐to‐CO process under different scenarios is calculated based on material balance. The prices of CO_2_ electrolyzer stacks, electricity, CO_2_, anode materials, and membrane were all referenced to the U.S. Department of Energy as well as the latest values reported in the literature.^[^ ^50^ ^]^ The cost of g‐NiN3 and Ag NPs are 0.57 and 71.4 $·g^−1^ (Supporting Information), respectively, both calculated with a catalyst loading amount of 1.5 mg·cm^−2^. The detailed material balance and CO_2_ electroreduction cost were provided in the Supporting Information, with no consideration of the crossover of CO_2_ from cathode to anode in this process. Initially, based on the parameters of g‐NiN3 in different scenarios of eCO_2_‐to‐CO, the cost inputs of each item of total investment cost or annual operating costs are obtained as shown in Figure 6b,c. Under the basic scenario of eCO_2_‐to‐CO achieved in this work, the cost input of the CO_2_ electrolyzer stacks for the plant construction exceeds $29 million, accounting for 52.0% of the total investment cost. The cost of balance‐of‐plant is ≈$18.63 million with about one‐third of the total investment cost, and the remaining investment cost of the PSA separation or the working capital both account for a relatively small proportion. With the improvement of eCO_2_‐to‐CO performance, the cost input for CO_2_ electrolyzer stacks decreases to ≈$8.75 million in the advanced scenario, which is nearly 70.0% lower than the cost in the basic scenario, accounting for ≈44.7% of the total investment cost. When the best scenario of eCO_2_‐to‐CO is realized, the cost of CO_2_ electrolyzer stack is only ≈$3.43 million with a proportion of 35.8% in the total investment cost, and the investment cost of other items has also decreased. Notably, the cost of CO_2_ electrolyzer stack is the main part of the total investment cost, varying significantly in different CO_2_ electroreduction scenarios.

Similarly, the annual operation cost for MEA replacement is significantly affected by the CO_2_ electroreduction scenarios. In the basic scenario, the annual cost of MEA replacement is as high as $6.36 million, accounting for 51.7% of the annual operation cost. However, the annual cost of MEA replacement decreases to ≈$750 thousand when the best scenario is applied, which accounts for only 14.5% of the annual operation cost. Electricity cost, maintenance cost, PSA separation cost, materials cost of CO_2_ and H_2_O, and cell compartment replacement cost have relatively little impact on the annual operation cost in different scenarios. Based on the above analysis, it was concluded that both the total investment cost and the annual operation cost in the construction of CO_2_ electrochemical plants can be realized a significant reduction with the advancement of CO_2_ electroreduction technology.

According to the above cost calculations, the CO production costs of CO_2_ electroreduction in g‐NiN3 or Ag NPs were further analyzed under different scenarios. As shown in Figure 6d, the CO production cost of CO_2_ electroreduction using Ag NPs is 1.23 $·kg^−1^ in the basic scenario. However, due to the lower cost of g‐NiN3 synthesized in this work compared to Ag NPs, the CO production cost of CO_2_ electroreduction using g‐NiN3 is 1.08 $·kg^−1^, which is only 0.28 $·kg^−1^ higher than the current market price (0.80 $·kg^−1^) of CO.^[^ ^50^ ^]^ With the improvement of the eCO_2_‐to‐CO performance, the CO production cost using g‐NiN3 or Ag NPs decreases to 0.51 or 0.68 $·kg^−1^ in the advanced scenario, respectively, falling below the market price of CO. As a result, when the eCO_2_‐to‐CO achieves a current density of 200.0 mA·cm^−2^ with 98% CO FE as well as 60% SPC of CO_2_, this technology shows promising profitability. Moreover, the CO_2_ electroreduction using either g‐NiN3 or Ag NPs can realize a 50% reduction in the CO production cost compared with the market price in the best scenario, showing a great potential for profitability.

To provide theoretical guidance for future CO_2_ electroreduction technology, a sensitivity analysis on different performance parameters of CO_2_ electroreduction was conducted based on the CO production cost realized by g‐NiN3 in the basic scenario, and the results are shown in Figure 6e. It is obvious that the variation of the current density has the greatest impact on the CO production cost while other conditions remain constant. In particular, CO_2_ electroreduction using g‐NiN3 at a current density of 61.4 mA·cm^−2^ corresponds to a CO production cost of 1.08 $·kg^−1^, whereas when the current density is reduced to 40.0 mA·cm^−2^, the CO production cost is as high as 1.49 $·kg^−1^. Conversely, the CO production cost dropped to 0.90 $·kg^−1^ with an increased current density of 80.0 mA·cm^−2^, demonstrating significant cost fluctuations due to changes in the parameter of current density. According to the analysis of total investment cost, the current density of CO_2_ electroreduction is directly related to the size of the CO_2_ electrolyzer stack. A lower current density value requires a larger CO_2_ electrochemical stack area and higher investment in CO_2_ electrochemical stack costs to achieve the same CO target yield. Since the cost of CO_2_ electrolyzer stack represents the highest proportion of the total investment cost in CO_2_ electrochemical plant construction, the parameter of current density has the greatest impact on the CO production cost.

In addition, the changes in MEA replacement years and electricity price parameters also have a great impact on CO production costs. For example, CO_2_ electroreduction using g‐NiN3 in the basic scenario with an MEA replacement period of 1 year and an electricity price of 0.03 $ kW h^−1^, the corresponding CO production cost is 1.08 $·kg^−1^. However, when extending the MEA replacement period to 0.5 years or setting the electricity price at 0.05 $·kW·h^−1^, the CO production cost reaches 1.44 or 1.21 $·kg^−1^, respectively, which are both significantly higher than the CO production cost of 1.08 $·kg^−1^. Conversely, if the MEA replacement period is set to 1.5 years or the electricity price is reduced to 0.01 $·kW·h^−1^, the corresponding CO production cost decreases to 0.96 or 0.95 $·kg^−1^, respectively. The CO_2_ price, CO FE, and SPC parameters have relatively little effect on CO production cost. Taking the CO_2_ price parameter as an example, when the CO_2_ price increases from 40 to 60 $·t^−1^, the CO production cost only rises from 1.08 to 1.11 $·kg^−1^, while the CO production cost decreases to 1.05 $·kg^−1^ with a CO_2_ price of 20 $·t^−1^. Hence, the variation in CO_2_ price only results in a mere fluctuation of 0.03 $·kg^−1^ compared to the CO production cost of 1.08 $·kg^−1^, indicating a minor impact of the CO_2_ price parameter on the CO production cost. In summary, future efforts to improve the current density of the system and the stability of MEA components, combined with renewable green electricity for reaction driving, are expected to maximize the profits of CO_2_ electroreduction technology.

## 3 Conclusion

In summary, this work developed a low‐cost and gram‐scale Ni SAC with a tri‐coordinated Ni‐N3 center for eCO_2_‐to‐CO. The g‐NiN3 delivers efficient CO_2_ conversion with 98.9% CO FE and outstanding durability in a 2 × 2 cm^2^ MEA cell. Moreover, the remarkable performance of 97.1% CO FE with a reaction current of 6.07 A, a 41.0% SPC of CO_2_ as well as a 43.1% EE of the system was simultaneously achieved for eCO_2_‐to‐CO in a large‐scale MEA reactor with an electrode area of 10 × 10 cm^2^, which is comparable to that of the precious Ag NPs and manifests as one of the most excellent eCO_2_‐to‐CO system. The TEA indicated that the eCO_2_‐to‐CO using g‐NiN3 can achieve a CO production cost of 1.08 $·kg^−1^ in the basic scenario, which is only 0.28 $·kg^−1^ higher than the market price of CO. This work reveals that future efforts should be made to achieve breakthroughs in the current density (up to 200 mA·cm^−2^) or MEA stability of eCO_2_‐to‐CO, and combining with green electricity driven is expected to realize profitable prospects.

## Conflict of Interest

The authors declare no conflict of interest.

## Supporting information



Supporting Information

## Data Availability

The data that support the findings of this study are available from the corresponding author upon reasonable request.
