# Supplementary material for: Gram‐Scale Preparation of Tri‐Coordinated Single‐Atom Catalysts for CO2 Electrolysis in Large‐Scale Membrane Electrode Assembly
Source: Adv Sci (Weinh). 2025 Mar 16;12(18):2500368. doi: 10.1002/advs.202500368 (PMC12079529; doi:10.1002/advs.202500368)
Supplement: Supplementary file 1 — Supporting Information [file ADVS-12-2500368-s001.docx]

**Supporting Information**

**Gram-scale Preparation of Tri-coordinated Single-atom Catalysts for CO_2_ Electrolysis in Large-scale Membrane Electrode Assembly**

Lei Yuan^1,2^, Xin Li^1^, Guilin Li^1^, Kuilin Peng^1^, Hongyu Zhang^1^, Shaojuan Zeng^1*^, Xiaofu Sun^4^, Xiangping Zhang^1,3^[[1]](#footnote-1)^*^

*^1^ Beijing Key Laboratory of Ionic Liquids Clean Process, State Key Laboratory of Multiphase Complex Systems, Key Laboratory of Green Process and Engineering, Institute of Process Engineering, Chinese Academy of Sciences, Beijing 100190, China*

*^2^* *School of Chemistry and Chemical Engineering, Henan Normal University, Xinxiang 453007, China*

*^3^ State Key Laboratory of Heavy Oil Processing, China University of Petroleum, Beijing 102249, China*

*^4^ Institute of Chemistry, Chinese Academy of Sciences, Beijing 100190, China*

**Contents**

1. **Materials and methods**
2. **Supplementary figures and tables**
3. **References**

**1. Materials and methods**

**1.1 Materials**

Carbon dioxide (CO_2_, 99.999%) was supplied by Tianhong (Langfang) Gas Co. Ltd, China. Potassium bicarbonate (KHCO_3_, 99.5%), potassium hydroxide (KOH, 85.0%), nickel chloride hexahydrate (NiCl_2_·6H_2_O, 98.0%) and isopropanol (ACS grade, 99.5%) were all purchased from Aladdin Biochem. Technol. (Shanghai) Co., Ltd, China. The commercial silver nanopowders (Ag NPs, 20-40 nm, 99.9%) were purchased from Alfa Aesar (China) Chemical Co., Ltd. Nafion D-521 dispersion and 1-butyl-3-methylimidazole chloride ([Bmim][Cl]) were provided by Hesen Electric (Shanghai) and Monils Chem. Eng. Sci. & Tech. (Shanghai) Co., Ltd, respectively. The materials, including carbon black (EC-600JD, 99.9%), gas diffusion layer (YLS-30T), anion exchange membrane (Sustainion X37-50), Ti felt (99.9%) and iridium oxide (IrO_2_, 99.9%) were all purchased from Sinero Technology (Suzhou) Co., Ltd, China. Concentrated hydrochloric acid (36~38 wt%) and concentrated nitric acid (65~68 wt%) were supplied by Beijing Chemical Factory, China. The Milli-Q water was obtained by a Direct-Pure UP system in our laboratory.

**1.2 Materials synthesis**

**Synthesis of metal ionic liquid.** The synthesis of 1-butyl-3-methylimidazole nickel tetrachloride ([Bmim]_2_[NiCl_4_]) was referred to our previously reported work. [Bmim][Cl] and NiCl_2_·6H_2_O were dispersed in water according to a molar ratio of 2:1, respectively, and reacted with continuous stirring at 70°C for 48 h. Finally, the reaction system was rotary evaporated and dried to obtain the target product.

**Activation of carbon black.** Carbon black (EC-600JD, 1.0 g) was dispersed in 100 mL of 8 M nitric acid solution and heated for reflux at 90°C for 6 h. After cooling to room temperature, the mixture was filtered and repeatedly washed with ultrapure water for further freeze-drying to obtain activated carbon black.

**Gram-scale synthesis of Ni-N3 SAC.** Activated carbon black (1.0 g) and 1-butyl-3-methylimidazolium nickel tetrachloride ([Bmim]_2_[NiCl_4_], 1.2 g) were dispersed in 500 mL H_2_O, respectively. Then the mixture was stirred continuously at 60°C for 10 h and dried by freeze-drying. The dried sample was spread uniformly in a 10×4×2 cm^3^ porcelain boat to ensure a thin spreading thickness and placed in a N_2_ atmosphere and pyrolyzed at 600°C for 2 h. After cooling to room temperature, the gram-scale target product was obtained by washing with 1 M hydrochloric acid solution and ultrapure water, centrifugation and freeze-drying.

**1.3 CO_2_ electrolysis in membrane electrode assembly (MEA)**

A MEA cell consisting of a cathode and anode chamber separated by a zero-gap membrane electrode was used for CO_2_ electrolysis. The zero-gap membrane electrode was made by pressing the cathode of g-NiN3@carbon paper (YLS-30T), anion exchange membrane of Sustainion X37-50, and anode of IrO_2_@Ti felt. The bipolar plates of the MEA cell were configured with a single serpentine channel, and the active area of the electrodes was 4 cm^2^. Humidified CO_2_ and 0.5 M KHCO_3_ were used as electrolytes for the cathode and anode, respectively. The CO_2_ electrolysis was performed under a two-electrode system without any reference electrode. The catalyst inks for the cathode and anode were sprayed on the side of substrates facing the Sustainion X37-50 membrane, and ensured a catalyst loading of 1.5 mg·cm^-2^. During the CO_2_ electrolysis, the humidified CO_2_ was continuously introducted into cathode chamber with a flow rate of 25 sccm, while the anode circulated 0.5 M KHCO_3_ at a flow rate of 25 sccm using a peristaltic pump. All electrochemical tests were carried out at room temperature without IR compensation.

A larger-scale MEA reactor with an active area of 100 cm^2^ was used for CO_2_ electrolysis to evaluate its potential for industrial applications. The testing methods were identical to that of the MEA cell described above. The main difference was that the bipolar plates of the large-scale MEA reactor were configured with four parallel serpentine channels to reduce pressure drop at the reactor inlet and outlet. Similarly, all electrochemical tests were carried out at room temperature without IR compensation.

**1.4 Characterization and products analysis**

Powder XRD measurements were recorded with a Rigaku Smartlab diffractometer with Cu Kα radiation (1.54 Å) operated at 45 kV and emission of 50 mA. The scattering range of 2*θ* was from 5˚ to 90˚, with a scanning rate of 15˚ min^-1^. SEM was performed using a ZEISS Gemini 300 electron microscopy operated at 3 kV. TEM was performed on FEI Talos F200X system. Aberration-corrected high-angle annular darkfield scanning transmission electron microscopy (AC-HAADF-STEM) was performed on Titan Cubed Themis G2300 system with a spherical aberration corrector. XPS was performed by a Thermo Fisher Scientific ESCALAB 250 Xi using an Al Kα (1486.6 eV). X-ray source under a pressure of 3🞨10^-7^ mbar, and the binding energy was referenced to the C 1s peak at 284.8 eV. Inductively coupled plasma-atomic emission spectrometry (ICP-AES) was carried on an ICPE-9000 to determine the metal content in single-atom catalysts. Ni K-edge analysis was performed with Si (111) crystal monochromators at the BL14W1 beamlines at the Shanghai Synchrotron Radiation Facility (SSRF) (Shanghai, China). Before the analysis at the beamline, samples were pressed into thin sheets with 1 cm in diameter and sealed using Kapton tape film. The XAFS spectra were recorded at room temperature using a 4-channel Silicon Drift Detector (SDD) Bruker 5040. Ni K-edge extended X-ray absorption fine structure (EXAFS) spectra were recorded in fluorescence mode. Negligible changes in the line-shape and peak position of Ni K-edge XANES spectra were observed between two scans taken for a specific sample. The XAFS spectra of these standard samples (NiPC, NiO, and Ni foil) were recorded in fluorescence mode. The spectra were processed and analyzed by the software codes Athena and Artemis.

Gaseous products (CO and H_2_) were collected into a sampling bag and quantified by Agilent 7890 Gas Chromatograph (GC) equipped with flame ionization detector (FID) and thermal conductivity detector (TCD). No other gaseous or liquid products were detected except for CO and H_2_ product. The quantification of gas products was determined by comparison with the standard curves. The standard curves for CO and H_2_, with above 0.999 correlation coefficient, were shown in Figure S8. After the analysis of GC, the products Faraday efficiency (FE) was calculated by the following Equation S1:

$FE\left(\%\right)=\frac{n_{\text{product}}ZF}{Q}\times100\%$ (S1)

where *Z* is the number of electrons to be transferred corresponding to the generated product, which is 2 for CO or H_2_. *F* means that the Faraday constant is 96485 C mol^-1^. *Q* represents the total electric charge consumed during the CO_2_ electroreduction. *n*_product_ represents the molar amount of the generated products.

**1.5 Single-pass conversion (SPC)**

The SPC of CO_2_ in the eCO_2_-to-CO was calculated using Equation S2 as shown below:

$\text{SPC (\%)=}\frac{\left(I\times FE\times t\times C_{e}\right)\div Z\div N_{A}}{V\div V_{m}\times t}$ (S2)

where *I* represents the total reaction current of the eCO_2_-to-CO, *t* is the duration of the electroreduction reaction, *C_e_* denotes the number of electrons corresponding to each coulomb of charge, which is 6.24×10^18^, *Z* indicates the number of electrons that need to be transferred to generate the target product, *N_A_* represents Avogadro constant of 6.02×10^23^, *V* is the flow rate of CO_2_ during the electroreduction process, and *V_m_* refers to the molar volume of gas under standard conditions, which is 22.4 L·mol^-1^.

**1.6 Energy efficiency (EE)**

The EE of the eCO_2_-to-CO system was calculated using Equation S3 as shown below:

$\text{EE (\%)=}\frac{\left(E_{OER}^{\circ}-E_{CO_{2}ER}^{\circ}\right)\times FE}{E}$ (S3)

Where *Eo OER* refers to the theoretical thermodynamic potential of the oxygen evolution reaction (OER) at the anode, which is 1.23 V. *Eo CO_2_ER* denotes the theoretical thermodynamic potential for the cathode CO_2_ electroreduction reaction aimed at a target product. This work primarily focuses on the eCO_2_-to-CO process with a theoretical thermodynamic reduction potential of -0.1 V. *E* represents the potential applied during the eCO_2_-to-CO process.

**1.7 Techno-economic assessment (TEA)**

Taking g-NiN3 as an example, we conduct a TEA of eCO_2_-to-CO in basic scenario. The production of CO is set at 50 t·d^-1^, and the FE_CO_ is 97.1%. Consequently, the total reaction current (*I*) required to achieve this output is:

| $I=\frac{50\times1000kg}{day}\times\frac{day}{86400s}\times\frac{1000g}{kg}\times\frac{mol}{28.01}\times2\text{e}^{-}\times\frac{96485C}{mol}\times\frac{1}{0.971}=4161668A$ | （S4） |
| --- | --- |

The SPC of CO_2_ is 41.0%. The required theoretical CO_2_ flow rate (*v*_1_) and the actual flow rate (*v*_2_) are as follows:

| $v_{1}=4161668A\times\frac{1}{2\text{e}^{-}}\times\frac{1}{96485C/mol}\times\frac{0.044kg}{mol}\times\frac{86400s}{day}=81987kg\cdot day^{-1}$ | （S5） |
| --- | --- |
| $v_{2}=81987kg\cdot day^{-1}\times\frac{1}{0.41}=199968kg\cdot day^{-1}$ | （S6） |

The required flow rate of H_2_O for the anode reaction is:

| $v_{H_{2}O}=4161668A\times\frac{1}{\text{4e}^{-}}\times\frac{1}{96485C/mol}\times\frac{0.018kg}{mol}\times\frac{86400s}{day}=16770kg\cdot day^{-1}$ | （S7） |
| --- | --- |

The total (CO_2_, CO, H_2_) flow rate (*v_T_*) at the cathode outlet of the CO_2_ electroreduction device is as follows (not considering CO_2_ crossover):

| $v_{CO_{2}}=199968kg\cdot day^{-1}\times\left(1-41\%\right)\times\frac{m^{3}}{1.98kg}\times\frac{day}{24h}=2483m^{3}\cdot h^{-1}$ | （S8） |
| --- | --- |
| $v_{CO}=\frac{50\times1000kg}{day}\times\frac{m^{3}}{1.14kg}\times\frac{day}{24h}=1827.5m^{3}\cdot h^{-1}$ | （S9） |
| $v_{H_{2}}=4161668A\times\left(1-97.1\%\right)\times\frac{1}{2\text{e}^{-}}\times\frac{1}{96485C/mol}\times\frac{0.002kg}{mol}\times\frac{3600s}{h}\times\frac{m^{3}}{0.0899kg}=72.5m^{3}\cdot h^{-1}$ | （S10） |
| $v_{T}=2483m^{3}\cdot h^{-1}+1827.5m^{3}\cdot h^{-1}+72.5m^{3}\cdot h^{-1}=4382.8m^{3}\cdot h^{-1}$ | （S11） |

Based on the total current (4161668 A) and current density (60.7 mA·cm^-2^), the area (*S*) of CO_2_ electrolyzer stack is calculated as:

| $S=\frac{4161668A}{60.7mA\cdot cm^{-2}}\times\frac{1000mA}{A}\times\frac{m^{2}}{10000cm^{2}}=6778.0m^{2}$ | （S12） |
| --- | --- |

The applied potential for g-NiN3 in the basic scenario is 3.0 V, so the required power P is:

| $P=3.0V\times4161668A\times\frac{W}{1000000MW}=12.5MW$ | （S13） |
| --- | --- |

According to the calculations of material balance and energy consumption, the following is the cost calculation of total investment cost with the stack price of 4300 $·m^2^ as reference^1^, so the cost of CO_2_ electrolyzer stack is:

| $6778.0m^{2}\times4300\$\cdot m^{2}=29145232.6\$$ | （S14） |
| --- | --- |

The cost of balance of plant is based on 39% of the cost of CO_2_ electrolyzer stack:

| $29145232.6\$\times\frac{0.39}{0.61}=18633837.2\$$ | （S15） |
| --- | --- |

The cost for PSA equipment is calculated based on gas handling capacity and scaling factors. The investment benchmark for the PSA equipment is set at $1989043. In this work, the scaling factor is taken as 0.7^1^, leading to the cost of PSA equipment investment being:

| $1989043\$\times\left(\frac{4382.8m^{3}\cdot h^{-1}}{1000m^{3}\cdot h^{-1}}\right)^{0.7}=5595900.5\$$ | （S16） |
| --- | --- |

The cost of working capital is calculated at 5% of the above investment cost.

| $\left(29145232.6\$+18633837.2\$+5595900.5\$\right)\times5\%=2668748.5\$$ | （S17） |
| --- | --- |

The design lifespan of the CO_2_ electroreduction plant is 20 years, operating for 350 days each year, with a nominal interest rate calculated at 10%. Therefore, the total investment cost (TIC) for this project is calculated as follows, and it is converted into the annual investment cost (AIC) over the project period as:

| $TIC=29145232.6\$+18633837.2\$+5595900.5\$+2668748.5\$=56043718.9\$$ | （S18） |
| --- | --- |
| $AIC=\frac{56043718.9\$\times10\%}{1-\left(1+10\%\right)^{-20year}}=6582874.2\$$ | （S19） |

The following is the calculation for the annual operating cost. The electricity price is calculated at 0.03 $·kW·h^-1^, the annual maintenance cost of the plant is estimated at 2.5% of the stack cost, and the PSA separation cost is calculated based on the consumption of 0.25 kW·h of electricity for the separation of 1 m^3^ gas. Therefore, the electricity cost, maintenance cost, and PSA separation cost are calculated as follows:

| $12.5MW\times\frac{1000kW}{MW}\times\frac{24h}{day}\times\frac{350day}{year}\times0.03\$\cdot kWh^{-1}=3146221.1\$$ | （S20） |
| --- | --- |
| $29145232.6\$\times2.5\%=728630.8\$$ | （S21） |
| $4382.8m^{3}\cdot h^{-1}\times0.25kWh\cdot m^{-3}\times0.03\$\cdot kWh^{-1}\times\frac{24h}{day}\times\frac{350day}{year}=276116.1\$$ | （S22） |

The cost of CO_2_ and H_2_O feedstocks in the annual operating costs is calculated as follows, where the prices of CO_2_ and H_2_O are calculated at 40 $·t^-1^ and 0.0054 $·gal^-1^, respectively:

| $81987kg\cdot day^{-1}\times\frac{t}{1000kg}\times40\$\cdot t^{-1}\times\frac{350day}{year}=1147815.5\$$ | （S23） |
| --- | --- |
| $16770kg\cdot day^{-1}\times\frac{0.2642gal}{kg}\times0.0054\$\cdot gal^{-1}\times\frac{350day}{year}=8373.9\$$ | （S24） |

The cell compartment is based on the replacement every 7 years, and the operating cost of cell compartment replacement is calculated at 15% of the stack cost, so the annual operating cost of cell compartment replacement is:

| $29145232.6\$\times15\%\times\frac{1}{7year}=624540.7\$$ | （S25） |
| --- | --- |

MEA is a critical component in CO_2_ electrolyzer stacks, with costs that include the anode and cathode catalyst materials as well as the membrane materials. The stability period for the catalyst and membrane materials is set at one year. The anode catalyst materials are based on commercial iridium oxide, and their prices along with those of the membrane materials are based on references, calculated at 50 $·g^-1^ and 180 $·m^-2^, respectively^1^. Since the g-NiN3 in this work is synthesized from the metal ionic liquid [Bmim]_2_[NiCl_4_] and carbon black, and each gram of carbon black and [Bmim]_2_[NiCl_4_] can achieve a yield of about 1 g of g-NiN3, a cost assessment reveals that the price of the [Bmim]_2_[NiCl_4_] is approximately 0.14 $·g^-1^ (1,000 ¥·kg^-1^, based on an exchange rate of 1 $ ≈ 7 ¥), while the price of carbon black is about 0.43 $·g^-1^ (150 ¥·50 g^-1^). Thus, the price of g-NiN3 is roughly 0.57 $·g^-1^, significantly lower than the price of commercial Ag NPs, which is approximately 71.4 $·g^-1^ (2500 ¥·5 g^-1^, Alfa Aesar 20~40 nm). In calculating the cost of replacing the MEA, the load for both cathode and anode catalyst materials is based on 1.5 mg·cm^-2^. Taking g-NiN3 as an example, the price of the MEA per square meter is:

| $\left(\frac{10000cm^{2}}{m^{2}}\times1.5mg\cdot cm^{-2}\times\frac{mg}{1000g}\times\left(0.57+50\right)\right)+180=938.6\$\cdot m^{-2}$ | （S26） |
| --- | --- |

The annual operating cost of the MEA replacement is:

| $938.6\$\cdot m^{-2}\times6778.0m^{2}=6361455.4\$$ | （S27） |
| --- | --- |

The above cost represents the annual operating costs for each item, so the total annual operating cost (TOC) is:

| $TOC=12293153.5\$$ | （S28） |
| --- | --- |

In summary, the total annual investment for the CO_2_ electrochemical plant is the sum of the annual investment cost (AIC) and the total annual operating cost (TOC).

| $ACC+TOC=6582874.2\$+12293153.5\$=18876027.7\$$ | （S29） |
| --- | --- |

Based on a 50 t·d^-1^ CO output of the plant, a total of 17500 t of CO can be produced per year, and the unit CO production cost can be calculated according to the total annual investment as follows:

| $\frac{18876027.7\$}{17500t}\times\frac{t}{1000kg}=1.08\$\cdot kg^{-1}$ | （S30） |
| --- | --- |

**2. Supplementary figures and tables**


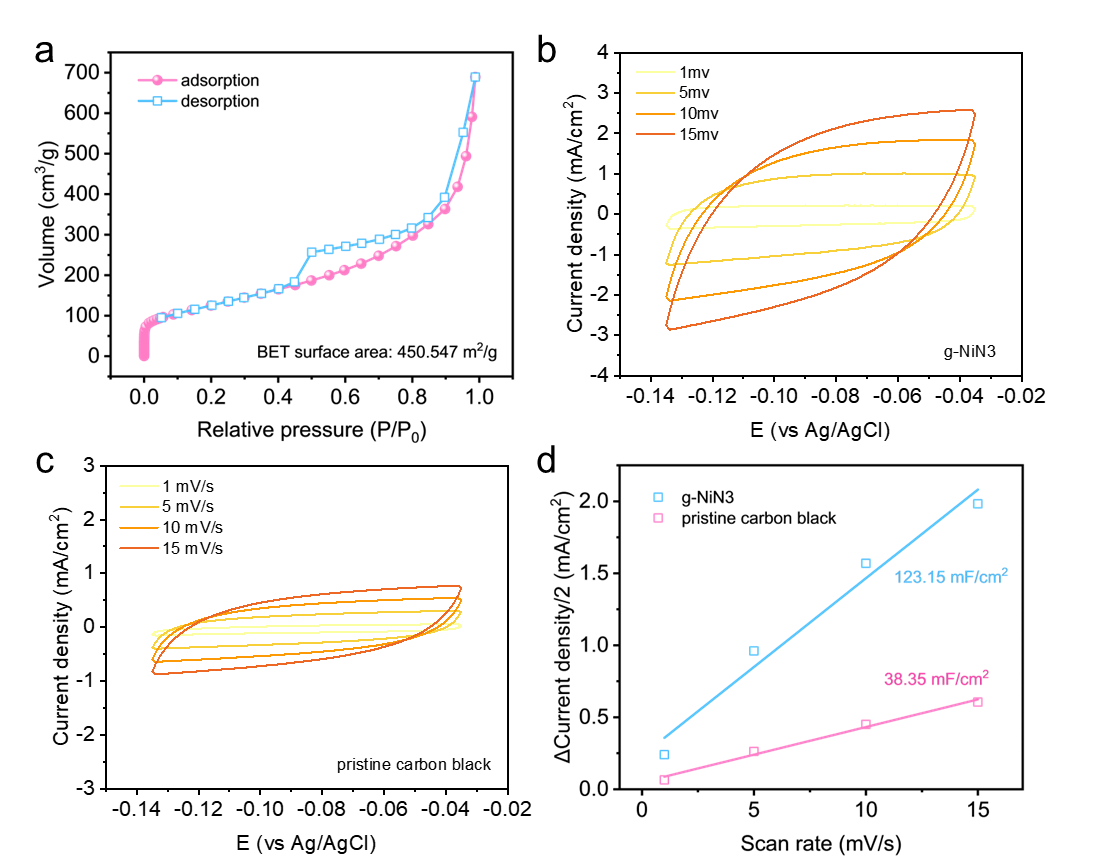


**Figure S1.** (a) N_2_ adsorption-desorption isotherms of g-NiN3. Cyclic voltammetry curves at different scan rates of (b) g-NiN3 and (c) pristine carbon black. (d) Comparison of the ECSA value between g-NiN3 and pristine carbon black.


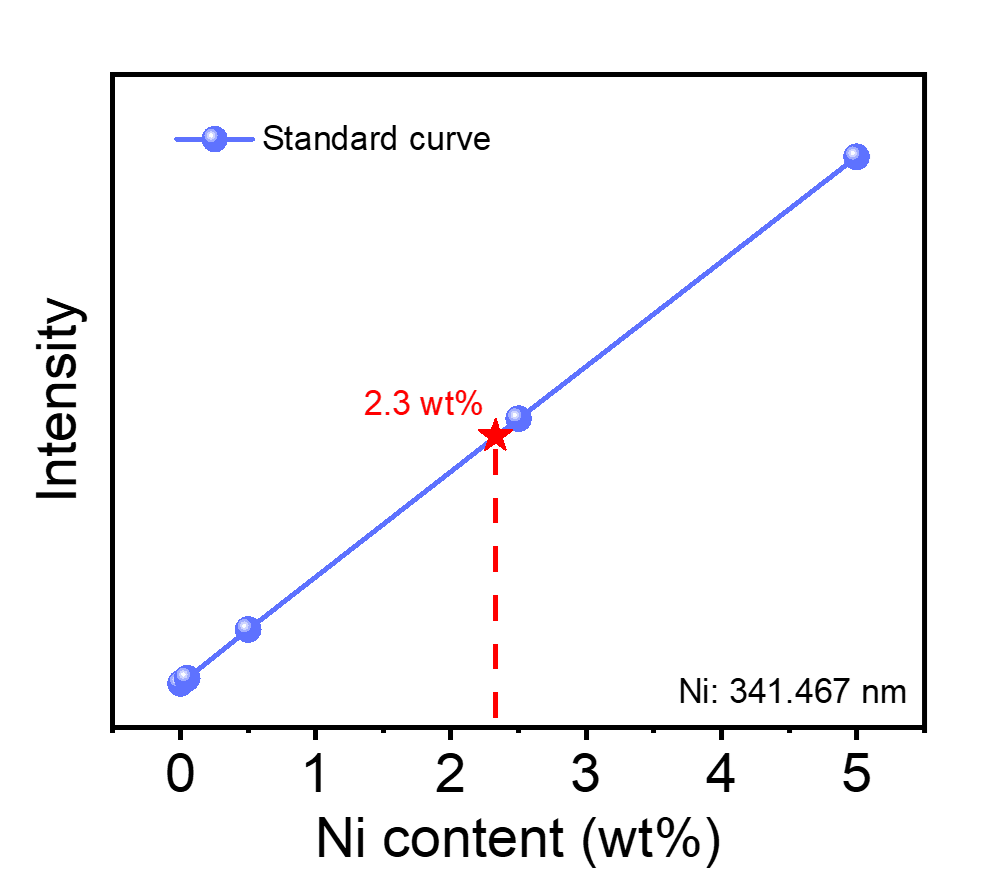


**Figure S2.** Ni content in g-NiN3 determined by ICP-AES.


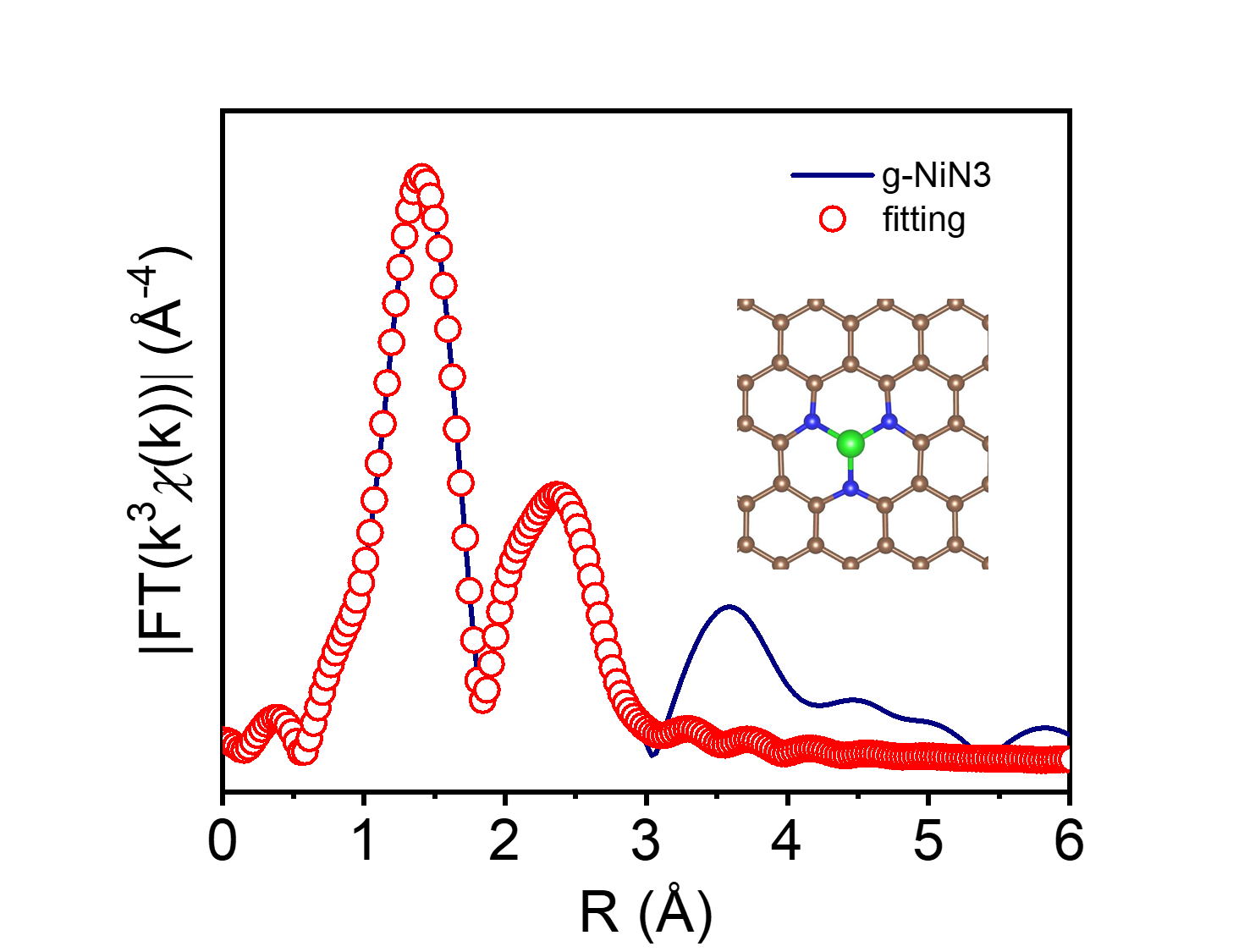


**Figure S3.** EXAFS fitting results and schematic configuration figure of the g-NiN3, where the balls in green, blue and brown represent Ni, N, and C atoms.

**Figure S4.** (a) Schematic diagram and (b) image of the MEA electrolysis system.


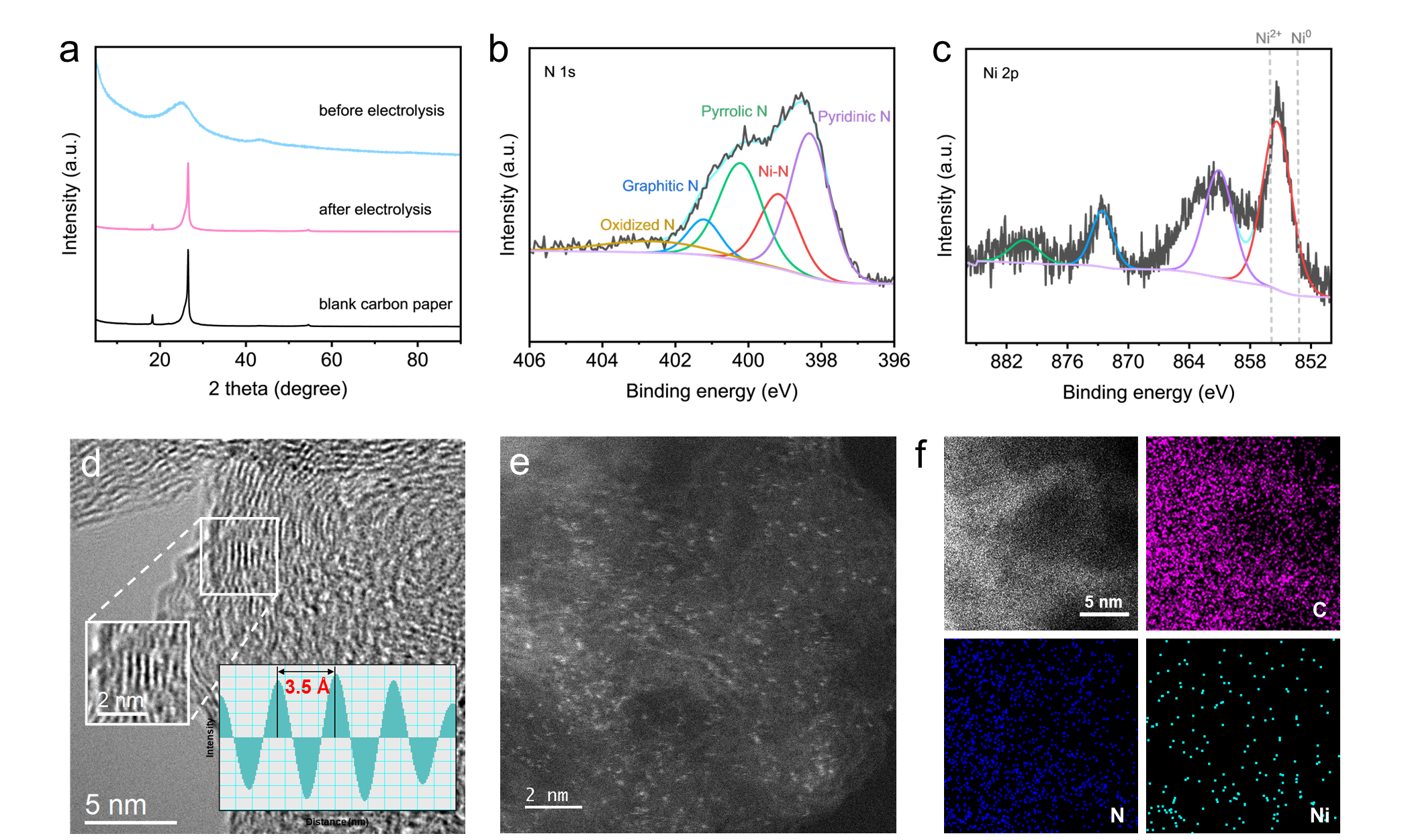


**Figure S5.** (a) XRD, (b) high-resolution N 1s spectrum, (c) high-resolution Ni 2p spectrum, (d) TEM, (e) HAADF-STEM and (f) element mapping results of g-NiN3 after 100 h electrolysis.

**Figure S6.** Process diagram of the CO_2_ electroreduction scale-up device.

**Figure S7.** The generated CO volume from continuous 10 h CO_2_ electrolysis in (a) Ag NPs and (b) g-NiN3, respectively.


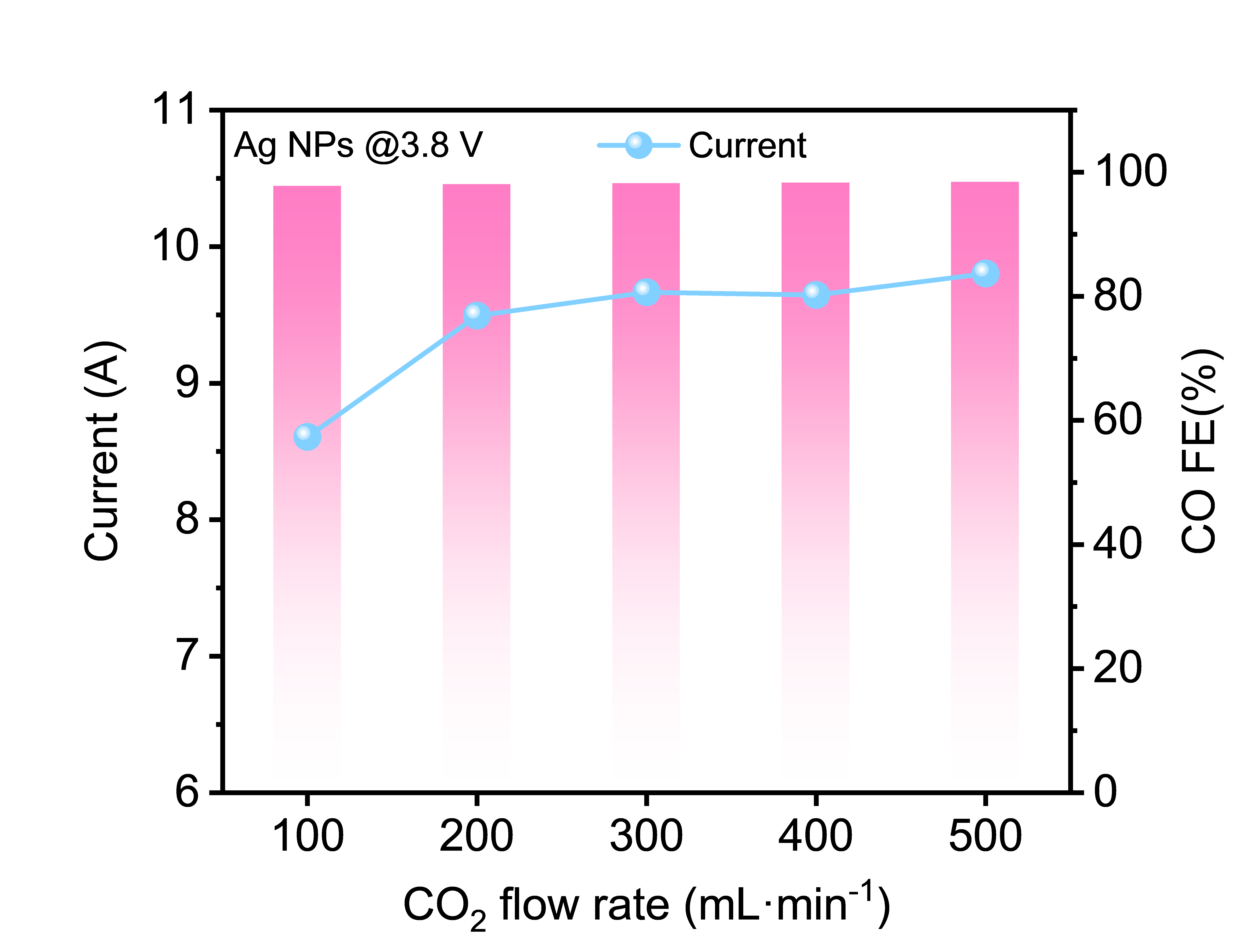


**Figure S8.** The effect of different CO_2_ flow rate on eCO_2_-to-CO performance in Ag NPs at 3.8 V.

**Figure S9.** Standard curves for CO (a) and H_2_ (b) with above 0.999 correlation coefficient.


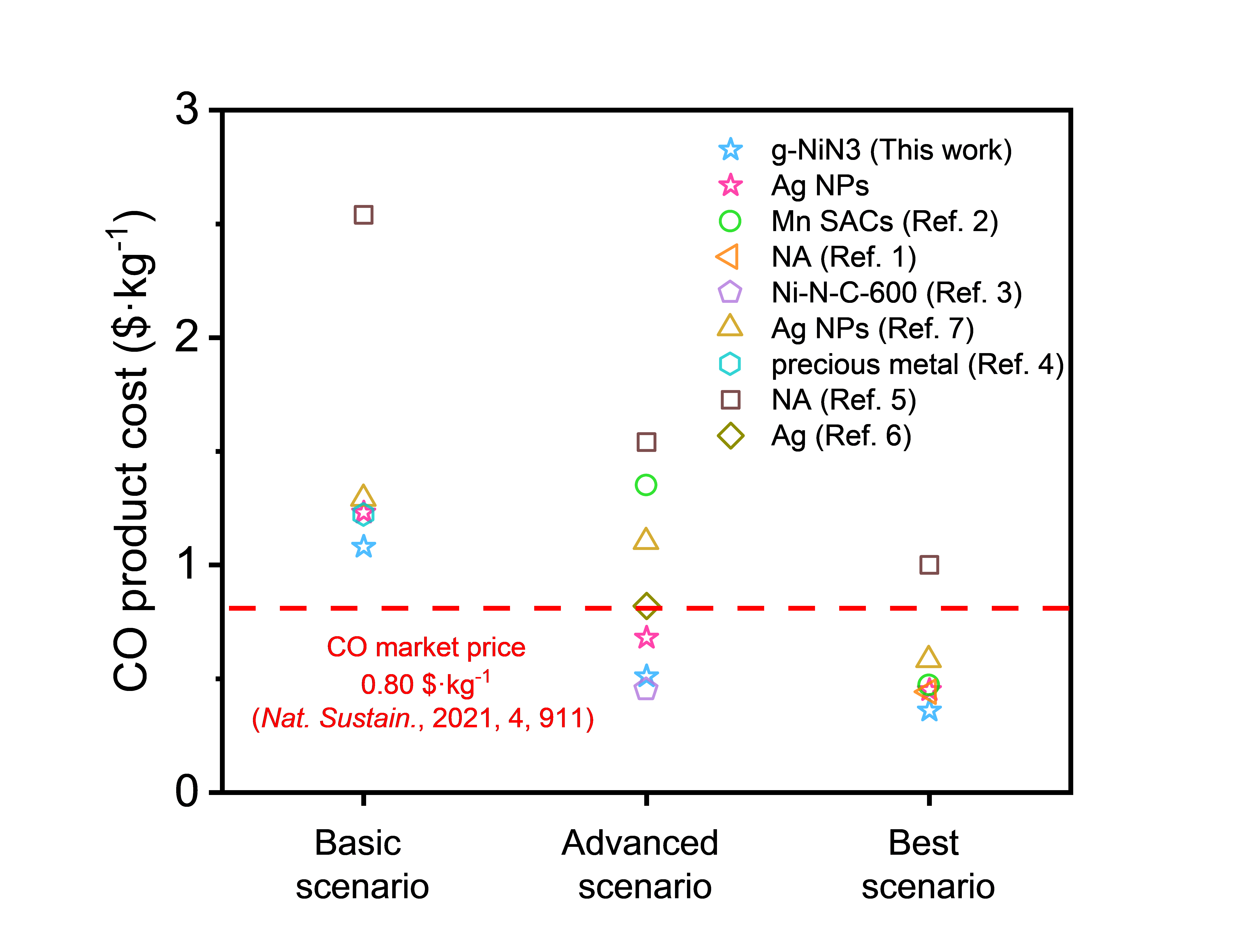


**Figure S10.** Comparison results of CO production cost of g-NiN3 with that of other catalysts in the literature^1-7^.

We summarized the data of the techno-economic assessment (TEA) for eCO_2_-to-CO in recent years and compared them with this work^1-7^, and the comparison results are shown in Figure S9.

Most of the literature reported that the precious metal such as Ag is usually used for eCO_2_-to-CO (*ACS Energy Lett.*, 2024, 9, 5550; *Green Chem.*, 2021, 23, 2397) and there are a few studies using non-precious metal single-atom catalysts (SACs) such as Ni SACs (*Appl. Catal. B Environ. Energy*, 2024, 358, 124396) and Mn SACs (*ACS Sustainable Chem. Eng.*, 2021, 9, 9045) for the TEA of eCO_2_-to-CO. According to the comparison results, the CO production cost by g-NiN3 is 1.08 $·kg^-1^ in the basic scenario, which is better than the value reported in other literature. Although the value is still higher than the market price (~0.80 $·kg^-1^) of CO, we can realize the competitive CO production costs with the improvement performance of eCO_2_-to-CO and the lower electricity price, e.g., 0.51 $·kg^-1^ in the advanced scenarios and 0.36 $·kg^-1^ in the best scenarios, which are superior to the most values reported in the literature. The above competitive CO production cost is ascribed to the low-cost synthesis of g-NiN3 in this work, which has a promising profitability prospect compared to the precious metal catalysts or other SACs used for eCO_2_-to-CO.

**Table S1.** Ni K-edge EXAFS fitting parameters of g-NiN3.

| Sample | Path | CN | R (Å) | σ^2^ (Å^2^) | ΔE_0_ | R factor |
| --- | --- | --- | --- | --- | --- | --- |
| Ni foil | Ni-N | 12 | 2.48 | 0.006 | 7.00 | 0.0009 |
| NiO | Ni-O | 6.0±0.1 | 2.09 | 0.005 | -1.70 | 0.0047 |
|  | Ni-O-Ni | 12.1±0.2 | 2.95 | 0.006 | -3.50 |  |
| NiPc | Ni-N | 4.0±0.1 | 1.90 | 0.003 | 6.60 | 0.0045 |
| g-NiN3 | Ni-N | 3.1±0.2 | 1.86 | 0.008 | -0.30 | 0.0042 |

CN: coordination numbers; R: bond distance; σ^2^: Debye-Waller factors (a measure of thermal and static disorder in absorber-scatter distances); ΔE_0_: the inner potential correction (the difference between the zero kinetic energy value of the sample and that of the theoretical model). R factor: goodness of fit Error bounds that characterize the structural parameters obtained by EXAFS spectroscopy were estimated as CN±20%, σ^2^±20%, R±0.03 Å.

**Table S2**. Comparison CO_2_-to-CO performance of g-NiN3 with reported electrocatalysts in a MEA cell^8-16^.

| Electrocatalysts | Active area (cm^2^) | FE_CO_ (%) | I (A) | SPC (%) | EE (%) | Ref. |
| --- | --- | --- | --- | --- | --- | --- |
| **g-NiN3** | **100** | **97.1** | **6.07** | **41.0** | **43.1** | **This work** |
| **Ag NPs (3.0 V)** | **100** | **99.2** | **2.24** | **15.5** | **44.0** |  |
| **Ag NPs (3.8 V)** | **100** | **96.0** | **8.97** | **60.0** | **33.6** |  |
| Ni-NCB | 100 | 90.0 | 8.00 | 11.3 | 42.8 | ***Joule***, 2019, 3, 265 |
| Co-CNTs-MW | 100 | 86.8 | 10.00 | 40.4 | 23.3 | ***Nat. Commun.***, 2023, 14, 1599 |
| Ag | 61 | 80.0 | 7.93 | 11.5 | 43.5 | ***ACS Energy Lett.***, 2019, 4, 1770 |
| e-Ag coral | 10 | 90.0 | 3.47 | 21.8 | 34.2 | ***Nano Energy***, 2020, 76, 105030 |
| Ag | 8 | 90.0 | 8.00 | 29.0 | 39.1 | ***Energy Environ. Sci.***, 2020, 13, 4098 |
| CoPc | 4 | 88.0 | 0.80 | 4.9 | 46.4 | ***Science***, 2019, 365, 367 |
| 3D-Ni-SAC | 4 | 92.5 | 1.60 | 20.6 | 35.2 | ***Nat. Synth.***, 2022, 1, 658 |
| Au/C | 3.2 | 85.0 | 1.60 | 9.5 | 37.7 | ***Energy Environ. Sci.***, 2019, 12, 2455 |
| Ag-MPL | 1.9 | 96.3 | 0.76 | 12.7 | 33.8 | ***ACS Energy Lett.***, 2019, 4, 2024 |

**Table S3**. Comparison of CO_2_ SPC of g-NiN3 with other eCO_2_-to-CO systems^9, 10, 12-23^.

| Electrocatalysts | Electrolyzer | Active area (cm^2^) | CO_2_ flow rate (mL∙min^-1^) | SPC (%) | Ref. |
| --- | --- | --- | --- | --- | --- |
| **g-NiN3** | **MEA** | **100** | **100 (3.0 V)** | **41.0** | **This work** |
|  |  |  | **100 (2.8 V)** | **29.0** |  |
|  |  |  | **200 (2.8 V)** | **21.0** |  |
|  |  |  | **300 (2.8 V)** | **14.2** |  |
|  |  |  | **400 (2.8 V)** | **10.7** |  |
|  |  |  | **500 (2.8 V)** | **8.5** |  |
| Ag | Flow cell | 495 | 200 | 18.5 | ***Chem. Eng. J.***, 2022, 450, 138378 |
| Ni-NCB | MEA | 100 | 500 | 11.3 | ***Joule***, 2019, 3, 265 |
| Ag | MEA | 61 | 433 | 11.5 | ***ACS Energy Lett.***, 2019, 4, 1770 |
| Ag | Flow cell | 10 | 90 | 18.6 | ***Nat. Catal.***, 2018, 1, 32-39 |
| Au/C | Flow cell | 10 | 50 | 23.7 | ***Nat. Commun.***, 2021, 12, 4943 |
| e-Ag coral | MEA | 10 | 100 | 21.8 | ***Nano Energy***, 2020, 76, 105030 |
| Ag | MEA | 6.25 | 50 | 9.0 | ***ACS Energy Lett.***, 2021, 6, 4291 |
| Ni_5_@NCN | MEA | 5 | 30 | 8.4 | ***ACS Appl. Mater. Interfaces***, 2022, 14, 7900 |
| Ag | MEA | 5 | 20 | 8.3 | ***Energy Technol.***, 2017, 5, 929-936 |
| CoPc | MEA | 4 | 100 | 4.9 | ***Science***, 2019, 365, 367 |
| 3D-Ni-SAC | MEA | 4 | 50 | 20.6 | ***Nat. Synth.***, 2022, 1, 658 |
| Au/C | MEA | 3.2 | 100 | 9.5 | ***Energy Environ. Sci.***, 2019, 12 ,2455 |
| Ag-MPL | MEA | 1.9 | 40 | 12.7 | ***ACS Energy Lett.***, 2019, 4, 2024 |
| Au/C | Flow cell | 1 | 50 | 3.2 | ***Nat. Catal.***, 2022, 5, 268-276 |

**Table S4**. Comparison of the EEs against SPCs in g-NiN3 with other eCO_2_-to-CO systems^8-23^.

| Electrocatalysts | SPC (%) | EE (%) | Ref. |
| --- | --- | --- | --- |
| **g-NiN3** | **41.0 (3.0V)** | **43.1** | **This work** |
|  | **29.0 (2.8V)** | **46.4** |  |
|  | **21.0 (2.8V)** | **46.6** |  |
|  | **14.2 (2.8V)** | **46.7** |  |
|  | **10.7 (2.8V)** | **46.7** |  |
|  | **8.5 (2.8V)** | **46.8** |  |
| Co-CNTs-MW | 40.4 | 23.3 | ***Nat. Commun.***, 2023, 14, 1599 |
| Au/C | 29.0 | 39.1 | ***Energy Environ. Sci.***, 2019, 12, 2455 |
| Ag | 18.5 | 27.9 | ***Chem. Eng. J.***, 2022, 450, 138378 |
| Ni-NCB | 11.3 | 42.8 | ***Joule***, 2019, 3, 265 |
| Ag | 11.5 | 43.5 | ***ACS Energy Lett.***, 2019, 4, 1770 |
| Ag | 18.6 | 22.6 | ***Nat. Catal.***, 2018, 1, 32-39 |
| Au/C | 23.7 | 22.6 | ***Nat. Commun.***, 2021, 12, 4943 |
| e-Ag coral | 21.8 | 34.2 | ***Nano Energy***, 2020, 76, 105030 |
| Ag | 9.0 | 32.0 | ***ACS Energy Lett.***, 2021, 6, 4291 |
| Ni_5_@NCN | 8.4 | 31.3 | ***ACS Appl. Mater. Interfaces***, 2022, 14, 7900 |
| Ag | 8.3 | 42.1 | ***Energy Technol.***, 2017, 5, 929-936 |
| CoPc | 4.9 | 46.4 | ***Science***, 2019, 365, 367 |
| 3D-Ni-SAC | 20.6 | 35.5 | ***Nat. Synth.***, 2022, 1, 658 |
| Au/C | 9.5 | 37.7 | ***Energy Environ. Sci.***, 2019, 12 ,2455 |
| Ag-MPL | 12.7 | 33.8 | ***ACS Energy Lett.***, 2019, 4, 2024 |
| Au/C | 3.2 | 37.8 | ***Nat. Catal.***, 2022, 5, 268-276 |

**Table S5.** The eCO_2_-to-CO performance of g-NiN3 and Ag NPs in different scenario.

| Parameter | Basic scenario | Advanced scenario | Best scenario |
| --- | --- | --- | --- |
| **g-NiN3** | | | |
| *j* (mA·cm^-2^) | 60.7 | 200 | 500 |
| FE_CO_ (%) | 97.1 | 98 | 100 |
| SPC (%) | 41.0 | 60 | 80 |
| **Ag NPs** | | | |
| *j* (mA·cm^-2^) | 89.7 | 200 | 500 |
| FE_CO_ (%) | 96.0 | 98 | 100 |
| SPC (%) | 60.0 | 60 | 80 |

**3. References**

1. H. Shin, K. U. Hansen and F. Jiao, *Nat. Sustain.*, 2021, **4**, 911-919.

2. J. Sun, H. Zhou, J. Xu, M. Wang, X. Liu, X. Huang, Y. Wang, S. Bai and Z. Huang, *Appl. Catal. B Environ. Energy*, 2024, **358**, 124396.

3. S. C. da Cunha and J. Resasco, *ACS Energy Lett.*, 2024, **9**, 5550-5561.

4. S. Lee, W. Choi, J. H. Kim, S. Park, Y. J. Hwang and J. Na, *Green Chem.*, 2023, **25**, 10398-10414.

5. M. Heßelmann, H. Minten, T. Geissler, R. G. Keller, A. Bardow and M. Wessling, *Adv. Sustainable Syst.*, 2023, **7**, 2300077.

6. J. Lee, W. Lee, K. H. Ryu, J. Park, H. Lee, J. H. Lee and K. T. Park, *Green Chem.*, 2021, **23**, 2397-2410.

7. F. Chang, G. Zhan, Z. Wu, Y. Duan, S. Shi, S. Zeng, X. Zhang and S. Zhang, *ACS Sustainable Chem. Eng.*, 2021, **9**, 9045-9052.

8. J. W. Sun, X. Wu, P. F. Liu, J. Chen, Y. Liu, Z. X. Lou, J. Y. Zhao, H. Y. Yuan, A. Chen, X. L. Wang, M. Zhu, S. Dai and H. G. Yang, *Nat. Commun.*, 2023, **14**, 1599.

9. Z.-Y. Wu, P. Zhu, D. A. Cullen, Y. Hu, Q.-Q. Yan, S.-C. Shen, F.-Y. Chen, H. Yu, M. Shakouri, J. D. Arregui-Mena, A. Ziabari, A. R. Paterson, H.-W. Liang and H. Wang, *Nat. Synth.*, 2022, **1**, 658-667.

10. W. H. Lee, Y. Ko, Y. Choi, S. Y. Lee, C. H. Choi, Y. J. Hwang, B. K. Min, P. Strasser and H. Oh, *Nano Energy*, 2020, **76**, 105030.

11. B. Endrődi, E. Kecsenovity, A. Samu, T. Halmágyi, S. Rojas-Carbonell, L. Wang, Y. Yan and C. Janáky, *Energy Environ. Sci.*, 2020, **13**, 4098-4105.

12. T. Zheng, K. Jiang, N. Ta, Y. Hu, J. Zeng, J. Liu and H. Wang, *Joule*, 2019, **3**, 265-278.

13. Z. Yin, H. Peng, X. Wei, H. Zhou, J. Gong, M. Huai, L. Xiao, G. Wang, J. Lu and L. Zhuang, *Energy Environ. Sci.*, 2019, **12**, 2455-2462.

14. R. Wang, H. Haspel, A. Pustovarenko, A. Dikhtiarenko, A. Russkikh, G. Shterk, D. Osadchii, S. Ould-Chikh, M. Ma, W. A. Smith, K. Takanabe, F. Kapteijn and J. Gascon, *ACS Energy Lett.*, 2019, **4**, 2024-2031.

15. S. Ren, D. Joulie, D. Salvatore, K. Torbensen, M. Wang, M. Robert and C. P. Berlinguette, *Science*, 2019, **365**, 367-369.

16. B. Endrődi, E. Kecsenovity, A. Samu, F. Darvas, R. V. Jones, V. Török, A. Danyi and C. Janáky, *ACS Energy Lett.*, 2019, **4**, 1770-1777.

17. L. Yuan, L. Zhang, J. Feng, C. Jiang, J. Feng, C. Li, S. Zeng and X. Zhang, *Chem. Eng. J.*, 2022, **450**, 138378.

18. Z. Liu, T. Yan, H. Shi, H. Pan, Y. Cheng and P. Kang, *ACS Appl. Mater. Interfaces*, 2022, **14**, 7900-7908.

19. J. Gu, S. Liu, W. Ni, W. Ren, S. Haussener and X. Hu, *Nat. Catal.*, 2022, **5**, 268-276.

20. K. Yang, M. Li, S. Subramanian, M. A. Blommaert, W. A. Smith and T. Burdyny, *ACS Energy Lett.*, 2021, **6**, 4291-4298.

21. M. C. O. Monteiro, M. F. Philips, K. J. P. Schouten and M. T. M. Koper, *Nat. Commun.*, 2021, **12**, 4943.

22. T. Haas, R. Krause, R. Weber, M. Demler and G. Schmid, *Nat. Catal.*, 2018, **1**, 32-39.

23. R. B. Kutz, Q. Chen, H. Yang, S. D. Sajjad, Z. Liu and I. R. Masel, *Energy Technol.*, 2017, **5**, 929-936.

1. *Corresponding authors: Tel./fax: +86-010-82544875.

   Email: [sjzeng@ipe.ac.cn](mailto:litao912@zzu.edu.cn) (Shaojuan Zeng), [xpzhang@ipe.ac.cn](mailto:renbz@zzu.edu.cn) (Xiangping Zhang) [↑](#footnote-ref-1)
